# Supplementary material for: Oxytocin induces the formation of distinctive cortical representations and cognitions biased toward familiar mice
Source: Nat Commun. 2024 Jul 25;15:6274. doi: 10.1038/s41467-024-50113-6 (PMC11272796; doi:10.1038/s41467-024-50113-6)
Supplement: Supplementary file 1 — Supplementary Information [file 41467_2024_50113_MOESM1_ESM.pdf]

Supplementary Information for

Oxytocin induces the formation of distinctive cortical representations and cognitions biased toward familiar mice

Wolf et al.

Includes:

Supplementary Figures 1-15
Supplementary Tables 1-4
Supplementary References

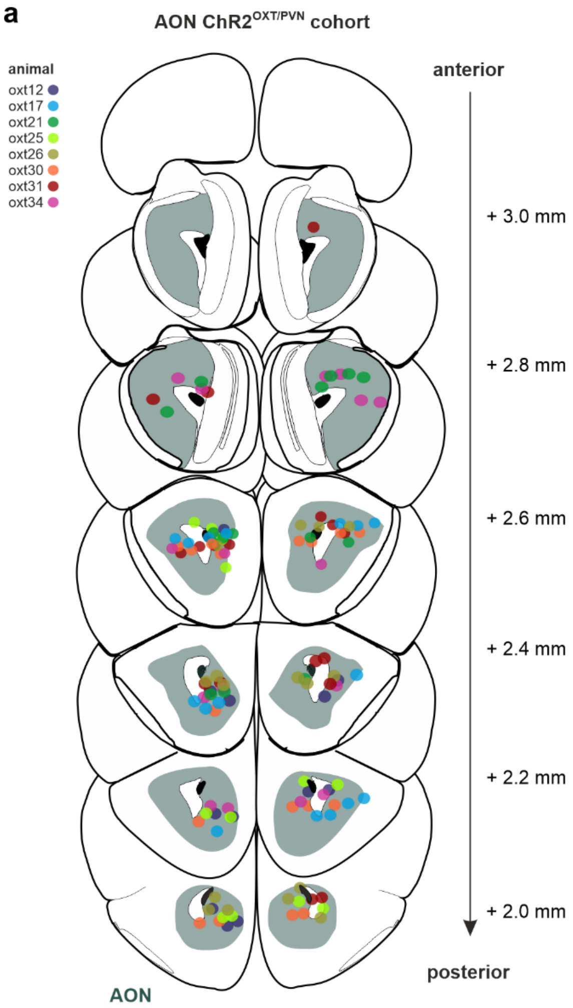
a
AON ChR2$^{OXT/PVN}$ cohort
animal
oxt12
oxt17
oxt21
oxt25
oxt26
oxt30
oxt31
oxt34
anterior
+ 3.0 mm
+ 2.8 mm
+ 2.6 mm
+ 2.4 mm
+ 2.2 mm
+ 2.0 mm
posterior
AON

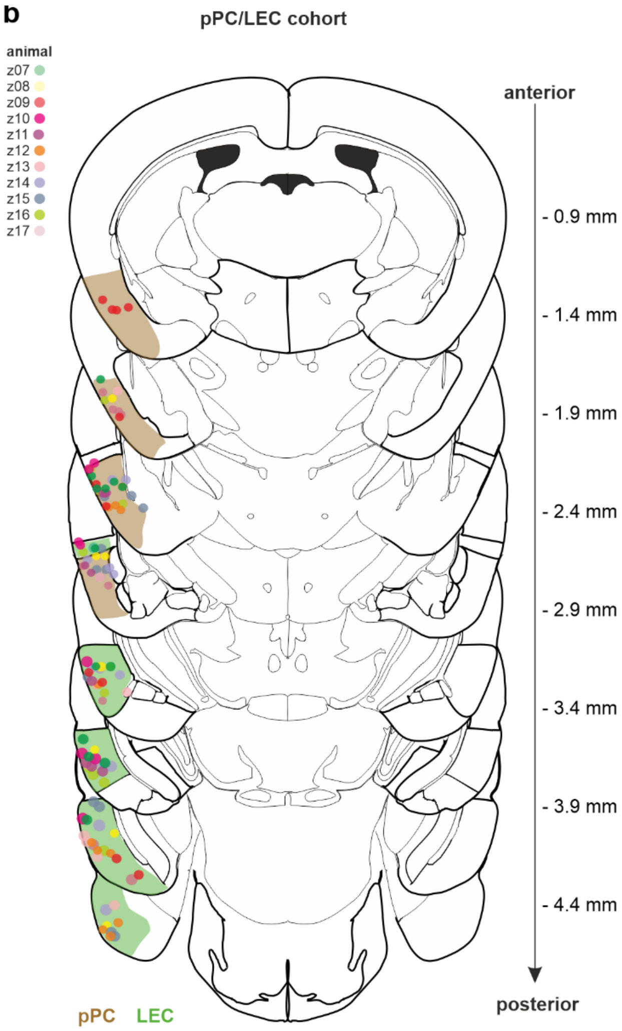
b
pPC/LEC cohort
animal
z07
z08
z09
z10
z11
z12
z13
z14
z15
z16
z17
anterior
- 0.9 mm
- 1.4 mm
- 1.9 mm
- 2.4 mm
- 2.9 mm
- 3.4 mm
- 3.9 mm
- 4.4 mm
posterior
pPC
LEC

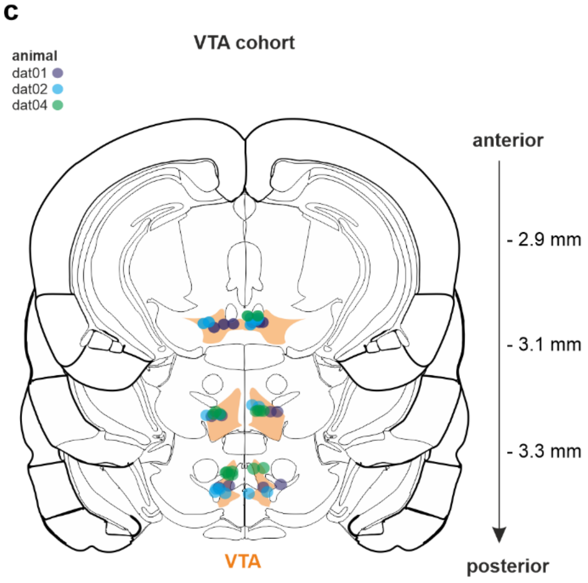
c
VTA cohort
animal
dat01
dat02
dat04
anterior
- 2.9 mm
- 3.1 mm
- 3.3 mm
posterior
VTA

Supplementary Fig. 1, related to Figs. 1, 2, 7, 8. Histological analysis of tetrode locations. a-c Histological confirmation of recording sites for tetrode recordings in the (a) AON (8 ChR2$^{OXT/PVN}$ mice), (b) the pPC (in brown) and LEC (in green; n = 11 mice) and (c) the VTA cohort (in orange, n = 3 mice). Tetrode tip locations mapped onto the Allen Reference Atlas – Mouse Brain with coordinates indicated relative to bregma. Animal identities are color-coded. The anatomical contours were drawn based on the Allen Reference Atlas – Mouse Brain, available from atlas.brain-map.org[1].

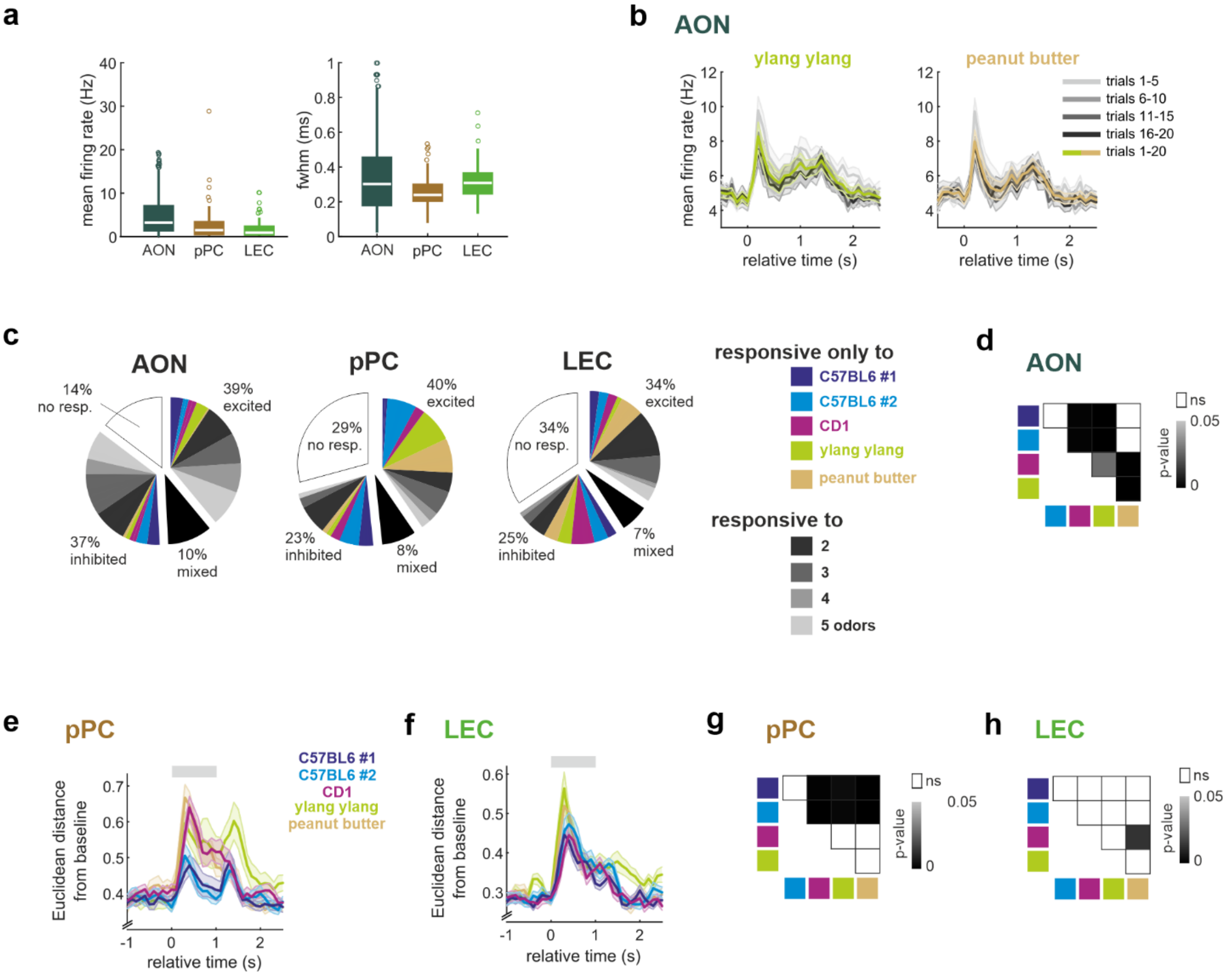

Supplementary Fig. 2, related to Fig. 1. Odor response tuning of single-units and population vector response features. a Average firing rate (left) and full width at half maximum (fwhm) of the unit spike waveforms (right) in the recorded regions. **b** Stability of the firing rate response to ylang ylang and peanut butter is shown for the AON in sequential blocks of 5 trials (grayscale, mean ± SEM, number of trials is indicated in the figure). Both odors show initial adaptation in the first block. After the first five trials, responses were stable in amplitude and shape throughout the session. **c** Neuronal response tuning of recorded regions. For every odor, the change in trial-averaged firing rate in the 1 s odor presentation window was tested for each neuron against baseline (two-sided Wilcoxon signed-rank test with Bonferroni correction for multiple comparisons). Significant increases in firing rate were termed odor-excited responses and significant decreases were termed odor-inhibited responses. Pie charts show fraction of units with excited or inhibited responses to a single odor (color-coded identity of odor), to multiple odors (grayscale), or with mixed response types (black). Mixed responses refer to units that show both significant odor-excited and odor-inhibited bins in the tested odor presentation period (1 s from odor onset). **d** The mean Euclidean distance from baseline was compared (0 to +1 s relative to odor onset); indicating differentiation in the population response amplitude (repeated-measures one-way ANOVA with post-hoc two-sided Tukey's test for multiple comparisons). P-values of the post-hoc tests were visualized as a heatmap, see also Supplementary Table 4. **e-f** The temporal evolution of the Euclidean distance from baseline of the population vector in the (e) pPC and (f) LEC (mean ± SEM, n = 20 trials per odor). Gray bar represents odor duration. **g-h** Same as (d) for (g) pPC and (h) LEC.

In this figure, test results are indicated as exact p-values or as a heatmap (see also Supplementary Table 4). Boxplots with a median indicated as horizontal line, the box edges indicating the 25th to 75th percentiles, a vertical line extending to the most extreme data points excluding outliers, and outliers plotted individually as circles. Source data are provided as a Source Data file.

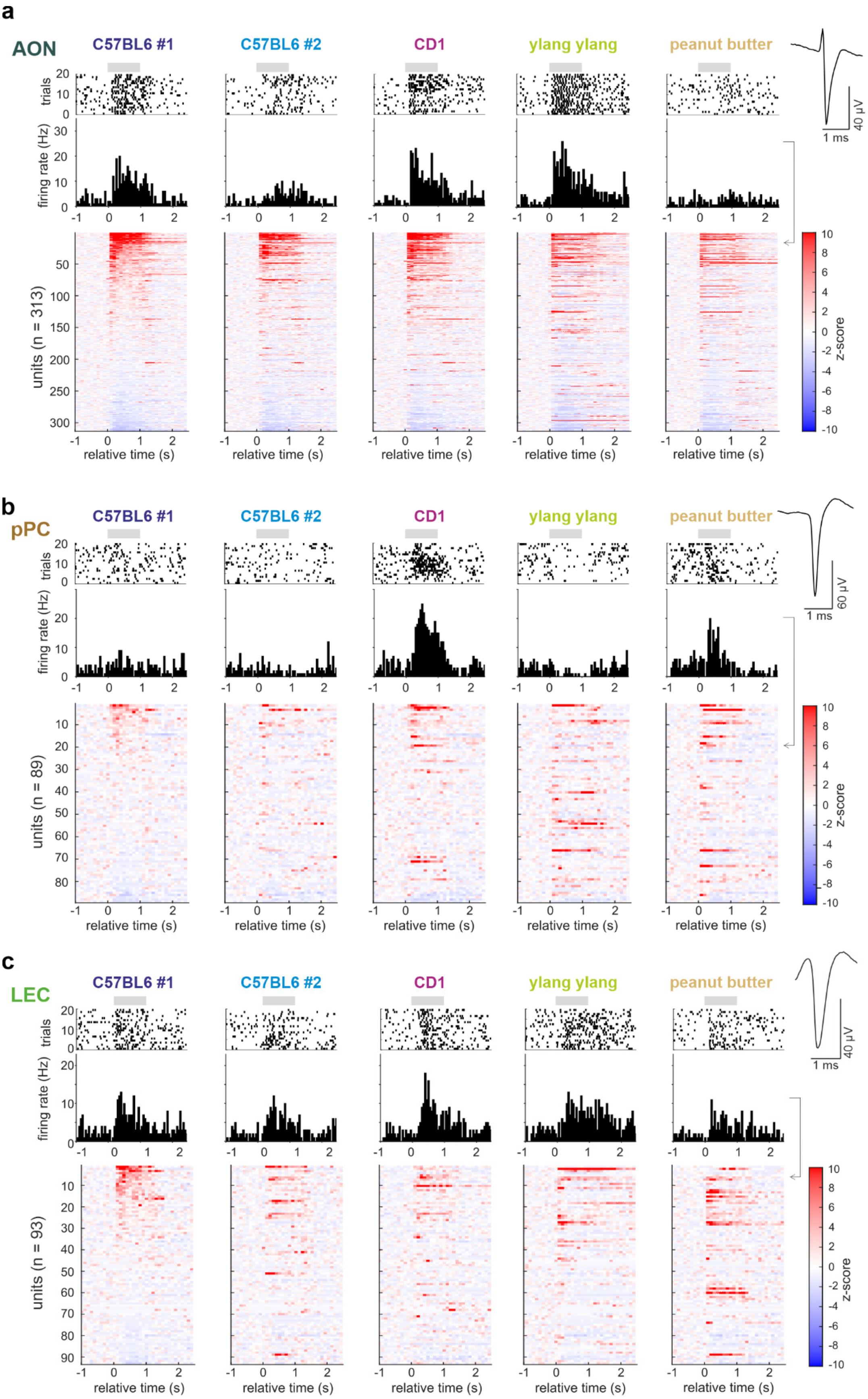
a
AON
C57BL6 #1
C57BL6 #2
CD1
ylang ylang
peanut butter
trials
firing rate (Hz)
units (n = 313)
relative time (s)
z-score
1 ms
40 µV
b
pPC
C57BL6 #1
C57BL6 #2
CD1
ylang ylang
peanut butter
trials
firing rate (Hz)
units (n = 89)
relative time (s)
z-score
1 ms
60 µV
c
LEC
C57BL6 #1
C57BL6 #2
CD1
ylang ylang
peanut butter
trials
firing rate (Hz)
units (n = 93)
relative time (s)
z-score
1 ms
40 µV

Supplementary Fig. 3, related to Fig. 1. Single-unit responses to social and non-social odors. a-c Top: example of single-unit firing activity in (a) AON, (b) pPC and (c) LEC in response to the different odors. Peri-stimulus time histogram (PSTH) and spike raster plots show stable odor responses. Right: Mean spike waveform of exemplary single-unit. Bottom: Odor responses for every recorded unit from AON, pPC and LEC are shown as the trial-averaged z-scored responses to the respective odor. The z-score normalizes the firing rate according to the baseline firing rate and variability (-4 to 0 s relative to odor onset). Units are sorted according to their mean response to the C57BL6 #1 during 0 to +1 s relative to odor onset.

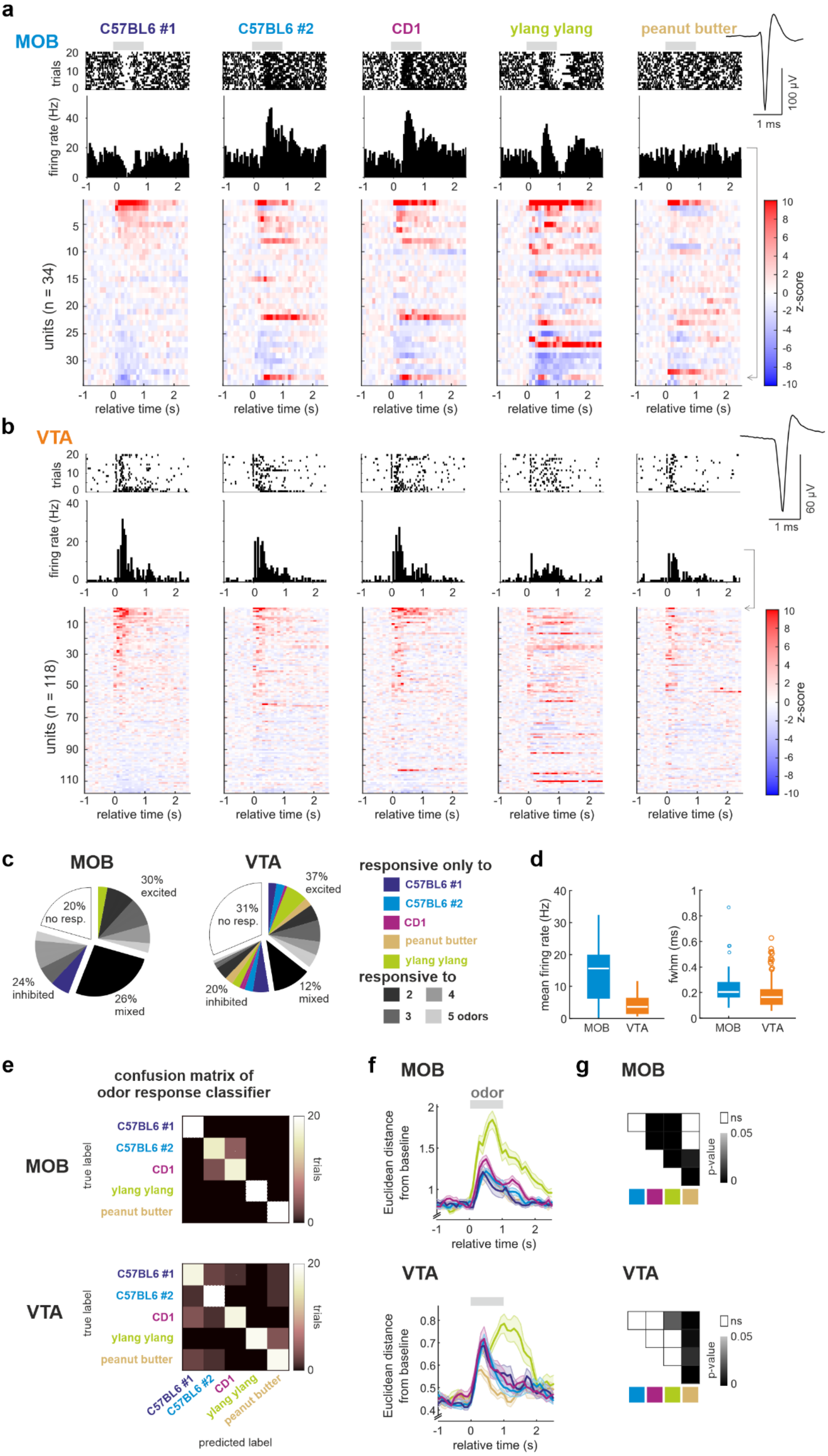
a
MOB
C57BL6 #1
C57BL6 #2
CD1
ylang ylang
peanut butter
trials
firing rate (Hz)
units (n = 34)
relative time (s)
100 µV
1 ms
z-score
b
VTA
units (n = 118)
60 µV
c
MOB
30% excited
20% no resp.
24% inhibited
26% mixed
VTA
37% excited
31% no resp.
20% inhibited
12% mixed
responsive only to
responsive to
2
3
4
5 odors
d
mean firing rate (Hz)
fwhm (ms)
e
confusion matrix of odor response classifier
true label
predicted label
f
odor
Euclidean distance from baseline
g
p-value
ns

Supplementary Fig. 4, related to Fig. 1. Representation of social odors in MOB and VTA. a-b Top: example of single-unit firing activity in (a) MOB and (b) VTA in response to the different odors. Peri-stimulus time histogram (PSTH) and spike raster plots show stable odor responses. Right: Mean spike waveform of exemplary single-unit. Bottom: Odor responses for every recorded unit from MOB and VTA are shown as the trial-averaged z-scored responses to the respective odor. Units are sorted according to their mean response to the C57BL6 #1 during 0 to +1 s relative to odor onset. **c** Neuronal response tuning of recorded regions. For every odor, the change in trial-averaged firing rate in the 1 s odor presentation window was tested for each neuron against baseline (two-sided Wilcoxon signed-rank test with Bonferroni correction for multiple comparisons). Significant increases in firing rate were termed odor-excited responses and significant decreases were termed odor-inhibited responses. Pie charts show fraction of units with excited or inhibited responses to a single odor (color-coded identity of odor), to multiple odors (grayscale), or with mixed response types (black). Mixed responses refer to units that show both significant odor-excited and odor-inhibited bins in the tested odor presentation period (1 s from odor onset). **d** Average firing rate (left) and full width at half maximum (fwhm) of the unit spike waveforms (right) in the recorded regions. (MOB: n = 34 units, VTA: n = 118 units) **e** The confusion matrices of linear decoders, which were trained to predict the odor identity of a single trial from the neuronal population activity in (top) MOB and (bottom) VTA, show high accuracy. Prediction accuracy was determined on trials, that were not included in the training dataset. Two-sided Fisher's exact test against decoder models trained with shuffled labels indicate prediction accuracy significantly above chance level (MOB: p = 4e-23; VTA: p = 1e-24). **f** The temporal evolution of the Euclidean distance from baseline of the population vector in the (top) MOB and (bottom) VTA (mean ± SEM, n = 20 trials per odor). Gray bar represents odor duration. **g** The mean Euclidean distance from baseline was compared in the (top) MOB and (bottom) VTA (0 to +1 s relative to odor onset); indicating differentiation in the population response amplitude (repeated-measures one-way ANOVA with post-hoc two-sided Tukey's test for multiple comparisons). P-values of the post-hoc tests were visualized as a heatmap, see also Supplementary Table 4.

In this figure, test results are indicated as exact p-values or as a heatmap (see also Supplementary Table 4). Boxplots with a median indicated as horizontal line, the box edges indicating the 25th to 75th percentiles, a vertical line extending to the most extreme data points excluding outliers, and outliers plotted individually as circles. Source data are provided as a Source Data file.

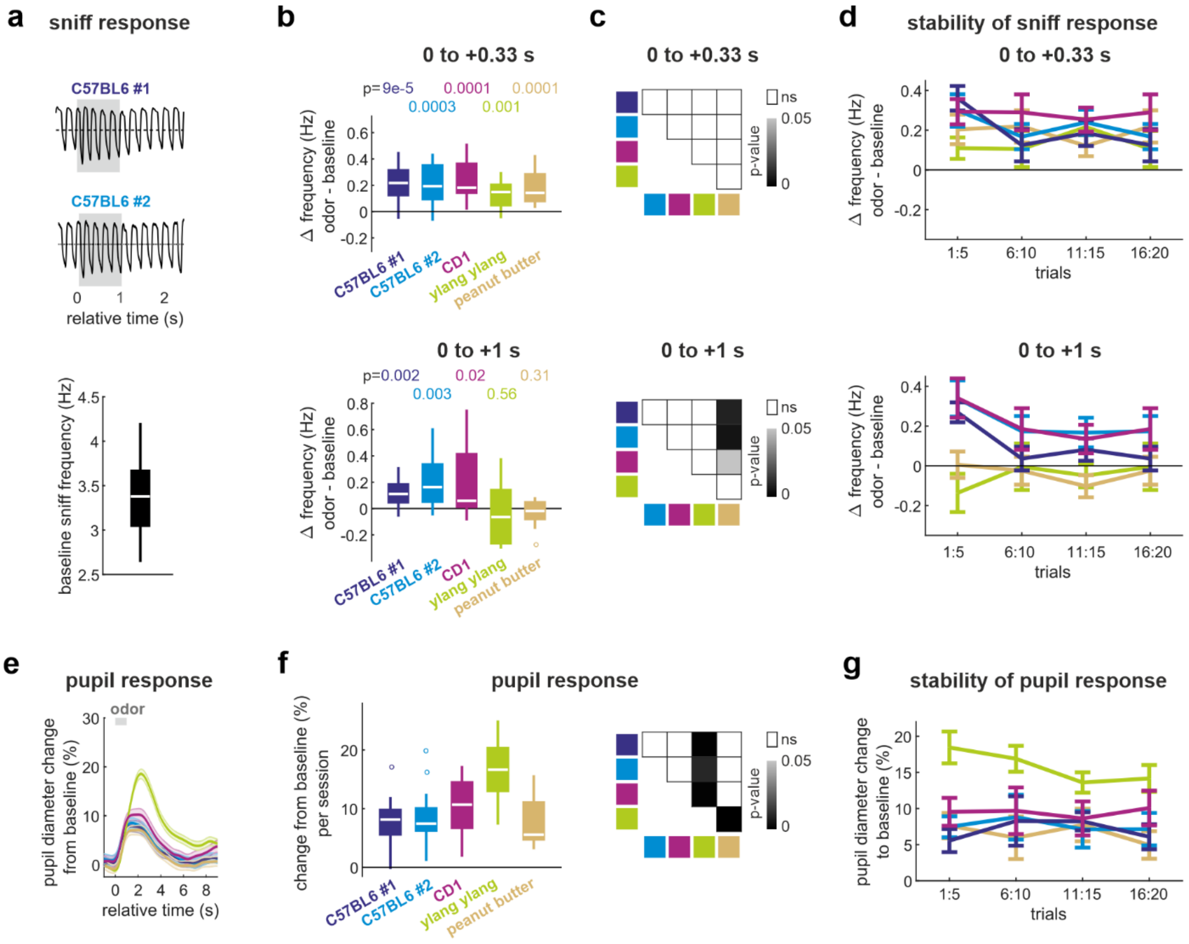

Supplementary Fig. 5, related to Fig. 1. Physiological responses to social and non-social odors. a (Top) Sniff response during odor presentation (gray box) in a single trial. (Bottom) Distribution of sniff frequencies without stimulus presentation (n= 13 animals with 1 session each). **b** Boxplot of the session-average change in sniff frequency from pre-stimulus baseline (top) in the first part (0 to +0.33 s) or (bottom) during the full window (0 to +1 s) of the odor presentation (n = 13 sessions, two-sided paired t-test). **c** P-values of pairwise comparisons of sniff frequency responses between odors during the (top) first part (0 to +0.33 s) or (bottom) during the full time (0 to +1 s) of odor presentation (repeated-measures one-way ANONVA with post-hoc two-sided Tukey's test for multiple comparisons; n = 13 animals with 1 session each). **d** Stability of sniff responses to each of the sampled odors is shown using the same metrics as in Fig. 1h for blocks of 5 consecutive trials as mean ± SEM during (top) the first part (0 to +0.33 s) or (bottom) the full time (0 to +1 s) of odor presentation. **e** Temporal evolution of the average pupil diameter response to each odor relative to baseline (mean ± SEM, n = 13 animals with 1 session each). **f** Left: Boxplot with session-average pupil diameter response between 1-3 s after odor onset relative to baseline. Right: p-values of the pairwise comparisons of odor responses (repeated-measures one-way ANOVA with post-hoc two-sided Tukey's test for multiple comparisons; n = 13 animals with 1 session each). **g** Stability of pupil responses to each of the sampled odors is shown using the same metrics as in (e) for blocks of 5 consecutive trials as mean ± SEM.
In this figure, test results are indicated as exact p-values or as a heatmap (see also Supplementary Table 4). Boxplots with a median indicated as horizontal line, the box edges indicating the 25th to 75th percentiles, a vertical line extending to the most extreme data points excluding outliers, and outliers plotted individually as circles. Source data are provided as a Source Data file.

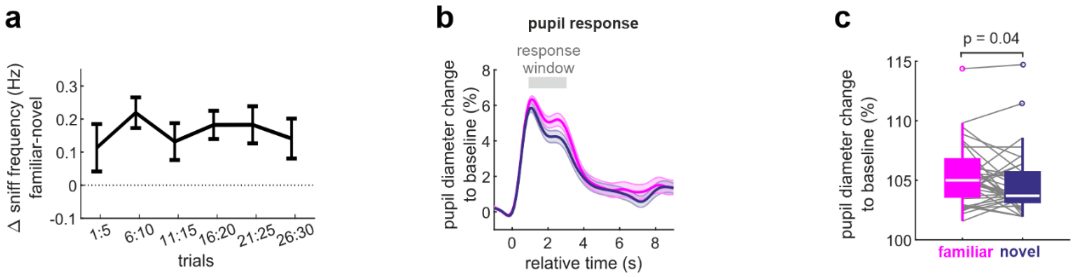

Supplementary Fig. 6, related to Fig. 2. Physiological responses to familiarity. a Stability of sniff frequency response is shown using the same metrics as in Fig. 2b for blocks of 5 consecutive trials. **b** The temporal evolution of the pupil response to odors from familiar and novel animals (mean percent change to baseline ± SEM, n = 38 sessions from 16 mice for control; additional pupil imaging sessions were performed to supplement the electrophysiology recording sessions). **c** The session-average pupil dilation response in the time window from +1 to +3 s after odor onset were compared between familiar and novel (two-sided paired t-test) and show greater pupil dilation in response to the familiar odor.

In this figure, test results are indicated as exact p-values (see also Supplementary Table 4). Boxplots with a median indicated as horizontal line, the box edges indicating the 25th to 75th percentiles, a vertical line extending to the most extreme data points excluding outliers, and outliers plotted individually as circles. Source data are provided as a Source Data file.

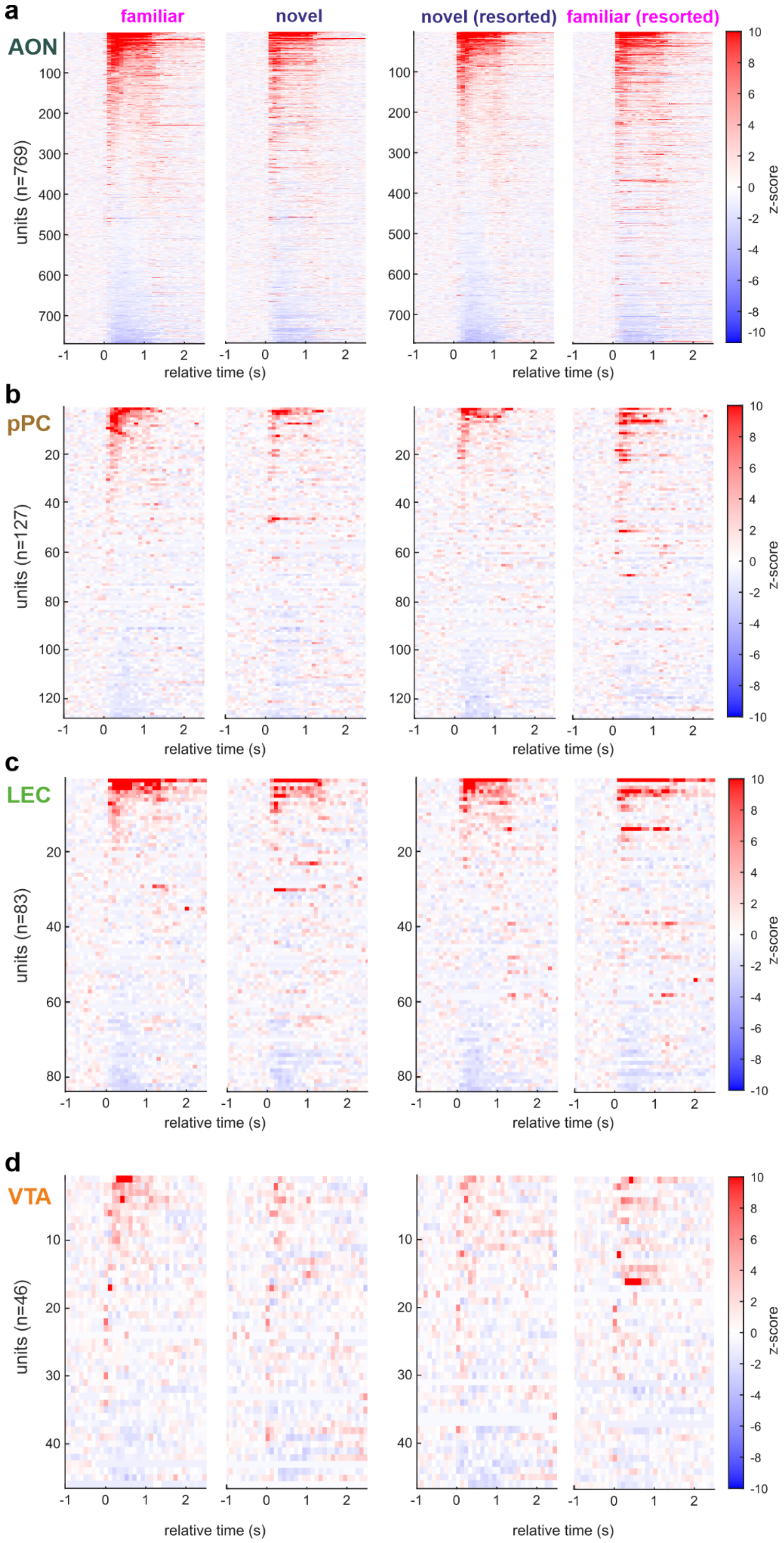
a
familiar
novel
novel (resorted)
familiar (resorted)
AON
units (n=769)
relative time (s)
z-score
b
pPC
units (n=127)
c
LEC
units (n=83)
d
VTA
units (n=46)

Supplementary Fig. 7, related to Fig. 2. Single-unit responses to familiar and novel social odors. a-d Trial-averaged z-scored response for each recorded unit to the different odor types: familiar and novel conspecifics. Units in the left two columns are sorted based on their mean response to the familiar odor during 0 to +1 s relative to odor onset. Responses in the right two columns are resorted according to the novel odor unit activity again during 0 to +1 s relative to odor onset.

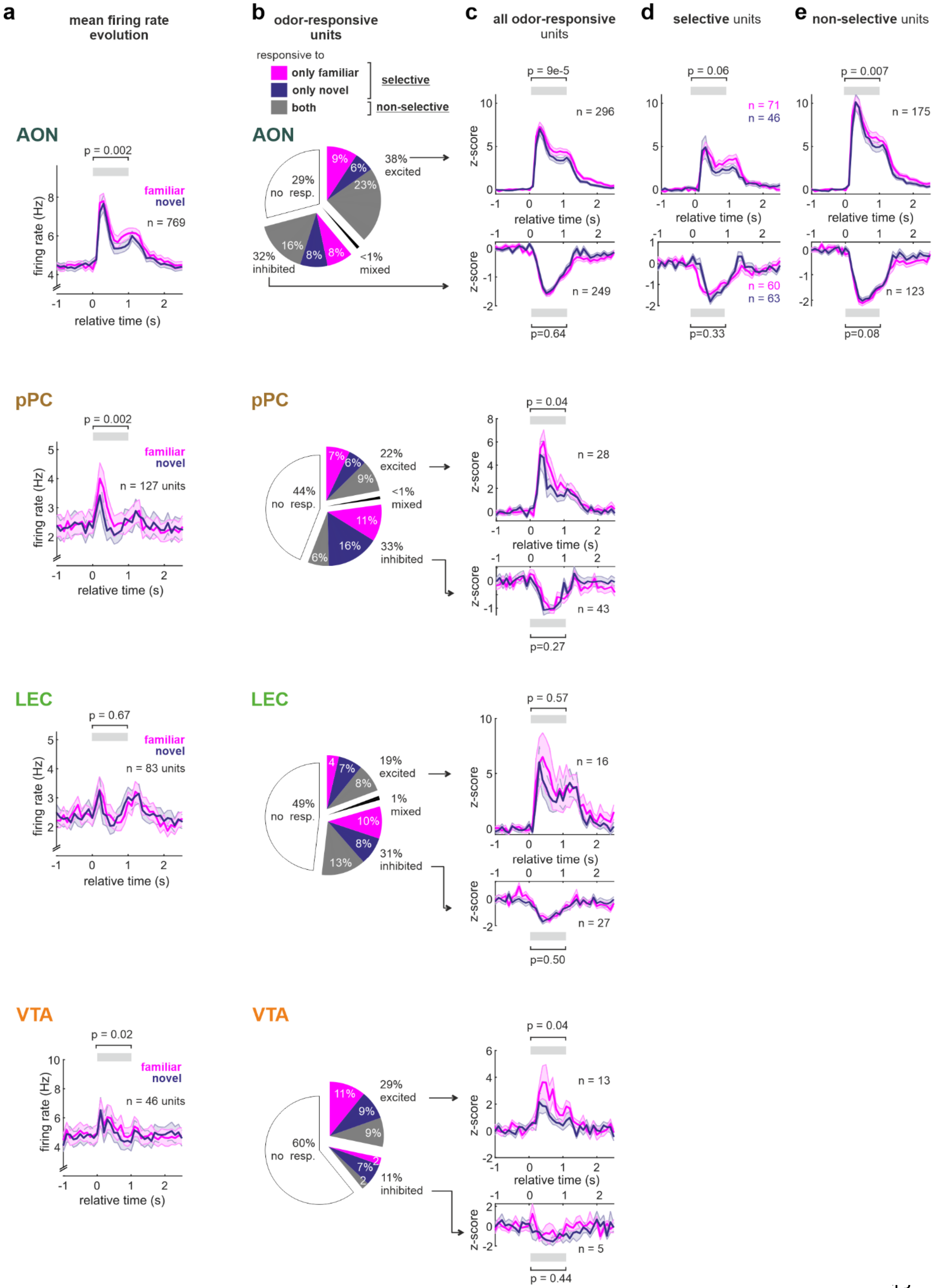
a
mean firing rate evolution
b
odor-responsive units
c
all odor-responsive units
d
selective units
e
non-selective units
responsive to
only familiar
only novel
both
selective
non-selective
AON
p = 0.002
familiar
novel
n = 769
firing rate (Hz)
relative time (s)
29%
no resp.
9%
6%
23%
38%
excited
<1%
mixed
16%
8%
8%
32%
inhibited
p = 9e-5
n = 296
z-score
n = 249
p=0.64
p = 0.06
n = 71
n = 46
n = 60
n = 63
p=0.33
p = 0.007
n = 175
n = 123
p=0.08
pPC
p = 0.002
n = 127 units
44%
no resp.
7%
6%
9%
22%
excited
<1%
mixed
11%
16%
6%
33%
inhibited
p = 0.04
n = 28
n = 43
p=0.27
LEC
p = 0.67
n = 83 units
49%
no resp.
4
7%
8%
19%
excited
1%
mixed
10%
8%
13%
31%
inhibited
p = 0.57
n = 16
n = 27
p=0.50
VTA
p = 0.02
n = 46 units
60%
no resp.
11%
9%
9%
29%
excited
2
7%
2
11%
inhibited
p = 0.04
n = 13
n = 5
p = 0.44

Supplementary Fig. 8, related to Fig. 2. Neuronal coding of familiarity a Temporal evolution of mean firing rate across all recorded units (mean ± SEM) in the (top to bottom) AON, pPC, LEC and VTA. Two-sided paired t-test comparing the mean firing rate, per unit, for familiar and novel odor stimuli were conducted for the 1 s odor duration window (number of units indicated). **b** Neuronal response tuning of recorded units. For every odor type, the trial-averaged firing rate response was tested against baseline (Two-sided Wilcoxon signed-rank test with Bonferroni correction for multiple comparisons). Significant increases in firing were termed odor-excited responses and significant decreases in spike rate were termed odor-inhibited responses. Mixed responses refer to units that show both significant odor-excited and odor-inhibited bins in the tested odor presentation period. Pie charts show fractions of units responding to one or more of the odors. **c** The temporal evolution of mean z-scored rate responses of all (top) odor-excited and (bottom) odor-inhibited units (mean ± SEM). Units were considered odor-excited if activated by at least one of the two odors and vice versa for odor-inhibited cells (Two-sided Wilcoxon signed rank-test comparing the mean z-scored response per unit. The corresponding p-values for the 1 s odor presentation window is shown above the respective line plots). **d** Similar to c, but for "selective" units that were significantly activated by either the familiar or the novel odor (Two-sided Wilcoxon rank sum test was used to compare the mean z-score response per unit. The corresponding p-values for the 1 s odor presentation window is shown above the respective line plots). **e** Similar to c, but for "non-selective" units that were significantly activated by both the familiar and novel odor. For the other brain regions, we did not split the responses as some subgroups become small (n = 3-12).
In this figure, test results are indicated as exact p-values (see also Supplementary Table 4). Source data are provided as a Source Data file.

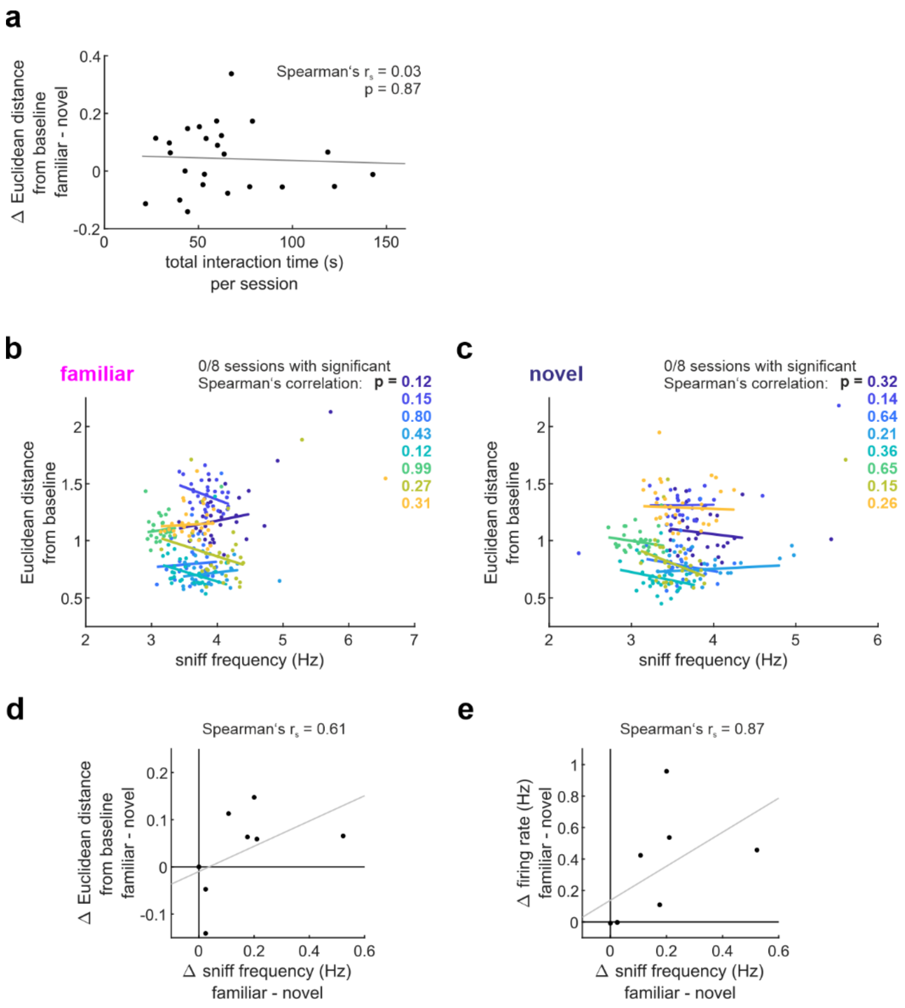

Supplementary Fig. 9, related to Fig. 2. Intra-individual correlations of behavioral readouts and neuronal activity. a The correlation of the memory strength (difference in Euclidean distance from baseline between familiar and novel) and the total interaction time during the freely-moving familiarization period shows no association (n = 8 animals, 3 sessions each). A linear fit was plotted for illustration of the slope. Spearman's rank correlation coefficient is indicated in the figure and was tested for significance. **b-c** For all AON sessions with simultaneous sniff recordings, we plotted the sniff frequency during (b) familiar and (c) novel odor presentations versus the mean Euclidean distance from baseline of that trial (0 to +1s relative to odor onset). We computed the trial-by-trial correlation for every session individually using Spearman's correlation coefficient and tested the significance of that correlation coefficient. We find no significant association of trial-by-trial sniff frequencies versus population response amplitude (p-values for every session indicated in the figure). Note that the reference lines for the correlation within the sessions was plotted for illustration without considering outliers, while the correlation coefficient was tested for significance considering all trials. **d-e** The correlation between memory strength (difference in (d) Euclidean distance from baseline and (e) average firing rate between familiar and novel) and sniff difference shows a positive correlation coefficient. This supports that per subject average sniff and neuronal responses correlated, consistent with the idea that familiarity is observed in either readout. (n = 8 sessions, same as b-c).
In this figure, test results are indicated as exact p-values (see also Supplementary Table 4). Source data are provided as a Source Data file.

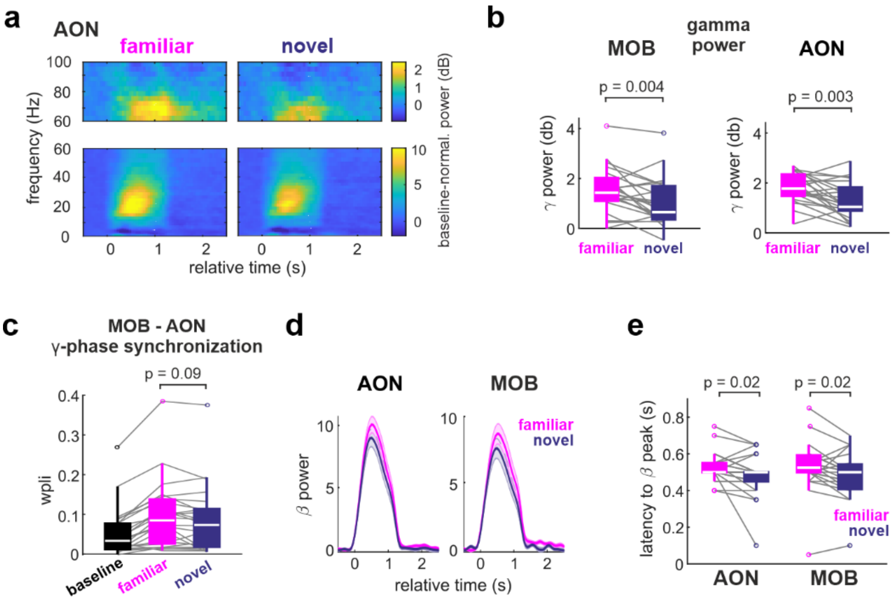

Supplementary Fig. 10, related to Fig. 3. Coordinated activity in the early olfactory loop during recognition of familiarity. a Odor-specific power spectrograms recorded in the AON were normalized to baseline and averaged across sessions (n = 23 sessions from 8 mice) and show a strong increase in oscillatory power in the β and γ bands upon odor presentation from the familiar and novel. **b** Within-session comparison of averaged γ power increase confirms stronger oscillatory responses to the familiar than novel smell in both regions (γ band time-frequency window: 60-80 Hz, 0.5 to +1.5 s relative to odor onset; n = 23 sessions, two-sided paired t-test). **c** Inter-regional phase synchronization of γ oscillations between the MOB and AON increases equally for both odors as compared to baseline (weighted phase lag index (wpli) per session compared with a two-sided Wilcoxon signed-rank test, n = 23 sessions). **d** Temporal evolution of β band power increase to the familiar and novel odor in the (left) AON and (right) MOB (mean ± SEM, n = 23 sessions). **e** The latencies to peak in the β band power per session in the (left) AON and (right) MOB for familiar versus novel smells were compared with a two-sided Wilcoxon signed rank-test (n = 23 sessions).

In this figure, test results are indicated as exact p-values (see also Supplementary Table 4). Boxplots with a median indicated as horizontal line, the box edges indicating the 25th to 75th percentiles, a vertical line extending to the most extreme data points excluding outliers, and outliers plotted individually as circles. Source data are provided as a Source Data file.

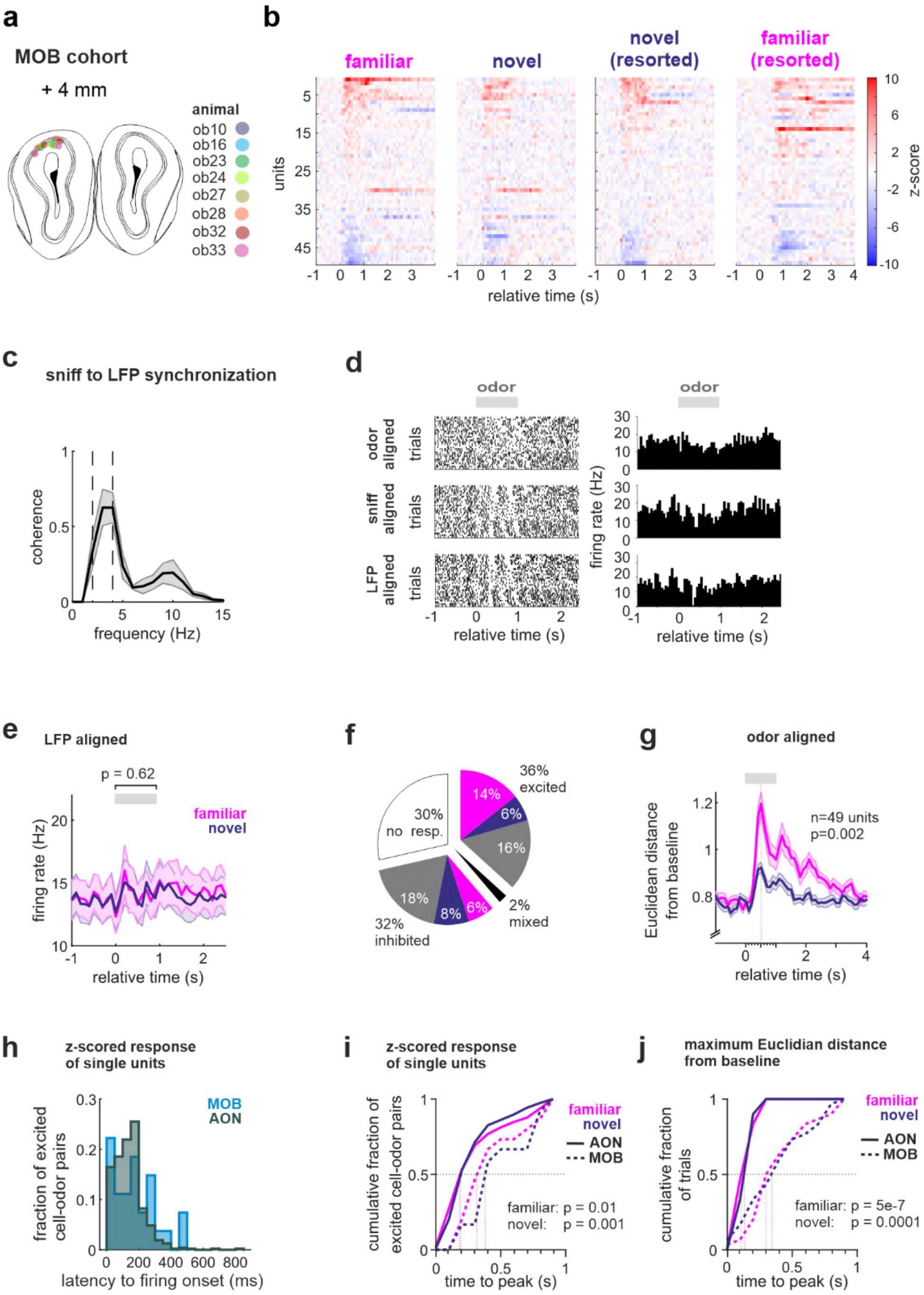

Supplementary Fig. 11, related to Fig. 4. MOB responses during recognition of familiarity. a Histological confirmation of recording sites for tetrode recordings in the MOB. Tetrode tip locations mapped onto the Allen Reference Atlas – Mouse Brain with coordinates indicated relative to bregma. Animal identities are color-coded. The anatomical contours were drawn based on the Allen Reference Atlas – Mouse Brain, available from atlas.brain-map.org[1]. **b** Trial-averaged z-scored response for each recorded unit to the different odor types: familiar and novel conspecifics. Units in the left two panels are sorted based on their mean response to the familiar odor during 0 to +1 s relative to odor onset. Responses in the right two panels are resorted according to the novel odor unit activity again during 0 to +1 s relative to odor onset. **c** The sniff signal shows a high coherence to the local field potential (LFP) in MOB in the frequency band around 2-4 Hz (mean ± SEM, n = 6 sessions). **d** Illustration of the effect of alignment to the ~3 Hz- LFP in comparison to sniff-alignment of an exemplary putative mitral cell. ~3 Hz-LFP-alignment has a comparable effect as sniff-alignment. Right: The response profile is only preserved in averaged trials after realignment (or after sniff-alignment) in the (left) raster plot and (right) PSTH. **e** Mean firing rate response of all recorded units after realignment (mean ± SEM, n = 49 units). **f** Neuronal response tuning of recorded

units. The trial-averaged firing rate response was tested against baseline (two-sided Wilcoxon signed-rank test with Bonferroni correction for multiple comparisons). Significant increases in firing were termed odor-excited responses and significant decreases in spike rate were termed odor-inhibited responses. Pie charts show fractions of units that are responsive to one or more of the odors. **g** The temporal evolution of the Euclidean distance from baseline of the population vector (mean ± SEM, n = 30 trials per odor) before realignment of spikes to the ~3 Hz-oscillation. The Euclidean distance from baseline was compared for the responses to familiar and novel emitters (0 to +1 s relative to odor onset); indicating a significantly stronger population response to the familiar odor (two-sided paired t-test). **h** The latency to firing onset of excited cell-odor pairs from MOB (n = 27 cell-odor pairs) and AON (n = 474 cell-odor pairs). The peak of the latency-to-onset distribution was earlier in MOB than AON, which is consistent with bottom-up flow of information. **i** Latency to peak response of odor-excited units for familiar (n = 15 and n = 250 units in the MOB and AON respectively) and novel (n = 12 and n = 224 units in the MOB and AON respectively) animals separately. Only units are included that show an odor-excited response to the given odor for MOB and AON. The latencies were compared using a two-sided Wilcoxon rank sum test, separately for familiar and novel. **j** Latency to peak response of the Euclidean distance from baseline per trial (n = 30 trials for familiar and novel respectively), without LFP-realignment, of the population vectors for MOB and AON (data from Fig. 2). The distributions of the two regions were compared using a two-sided Wilcoxon rank sum test separately for responses to familiar and novel animals.
In this figure, test results are indicated as exact p-values (see also Supplementary Table 4). Source data are provided as a Source Data file.

a

Mouse	1	2	3	4	5	6	
ChR2+	49	35	17	44	151	56	
α-OXT+	107	71	50	251	195	68	
ChR2+/α-OXT-	2	5	2	1	11	6	
ChR2-/α-OXT+	60	41	35	208	55	18	
ChR2+/α-OXT+	47	30	15	43	140	50	
	↓	↓	↓	↓	↓	↓	**Mean**
sensitivity (%)	44	42	30	17	72	74	**46.5**
specificity (%)	96	86	88	98	93	89	**91.7**

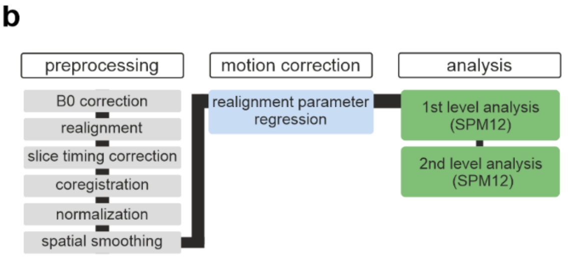

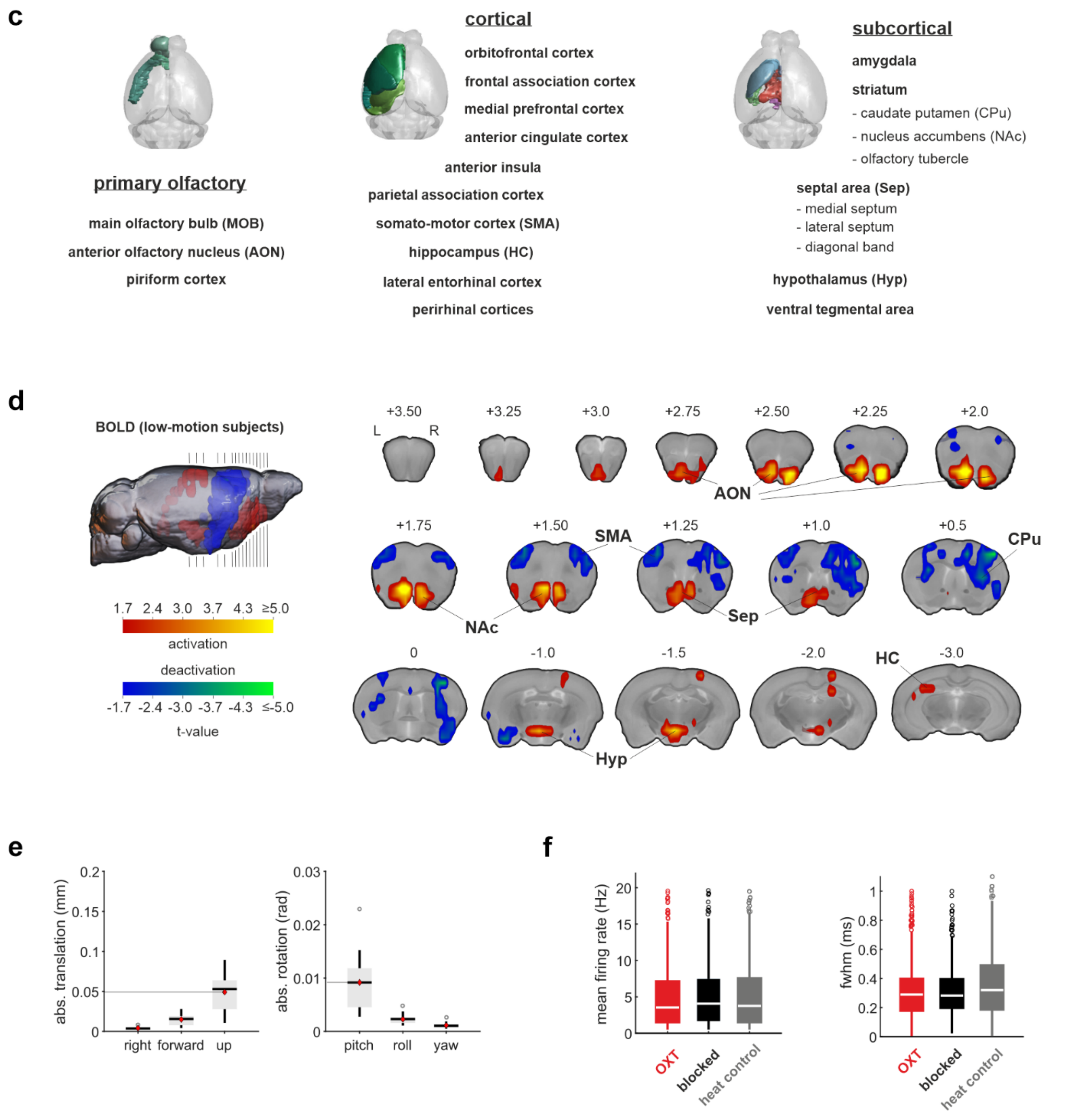

Supplementary Fig. 12, related to Figs. 5 and 6. Oxytocin neurons recruit AON activity in awake mice. a Histological characterization of the conditional expression of ChR2:mCherry in ChR2$^{OXT/PVN}$ mice (n = 6 mice). Sensitivity is defined as the fraction of ChR2:mCherry-positive cells out of all OXT-immunoreactive cells. Specificity is defined as the fraction of ChR2:mCherry-positive and also OXT-immunoreactive cells out of all ChR2:mCherry-positive cells. **b** fMRI preprocessing and analysis pipeline. At the single-

subject level, the realignment parameters for all translational and rotational directions were computed as nuisance regressors in the general linear regression to account for individual volume motion. **c** Parcellated network of structural regions in the social odor network mask broadly sorted into three categories (from left to right): primary olfactory, cortical and subcortical brain regions. The visualization was created with an in-house software[2] based on the Allen Reference Atlas – Mouse Brain, available from atlas.brain-map.org[1]. **d** The activation pattern of BOLD responses to OXT neuron activation of Fig. 5c was preserved when only testing the animals with event-related motion below the median of the cohort. Event-related motion was calculated at laser onset (average of two consecutive image volumes coinciding with stimulus onset). To illustrate the pattern of BOLD responses in this sub-sample (n = 11 sessions), the statistical threshold was lowered to $p < 0.05$, with $k > 50$ cluster size thresholding. **e** The absolute values of the first derivate of the realignment parameters were averaged within session (n = 23 mice, one session per mouse). Red diamonds indicate the mean across sessions, bars indicate the median, the box edges indicating the 25th to 75th percentiles, a vertical line extending to the most extreme data points excluding outliers, and outliers plotted individually as circles. **f** Average firing rates per session (left) and fwhm of spike waveforms (right) of AON single-units in the boosted OXT release and corresponding control conditions (n = 679 units in the OXT condition, n = 374 in the blocked control and n = 326 units in the heat control condition). Boxplots with a median indicated as horizontal line, the box edges indicating the 25th to 75th percentiles, a vertical line extending to the most extreme data points excluding outliers, and outliers plotted individually as circles.
Source data are provided as a Source Data file.

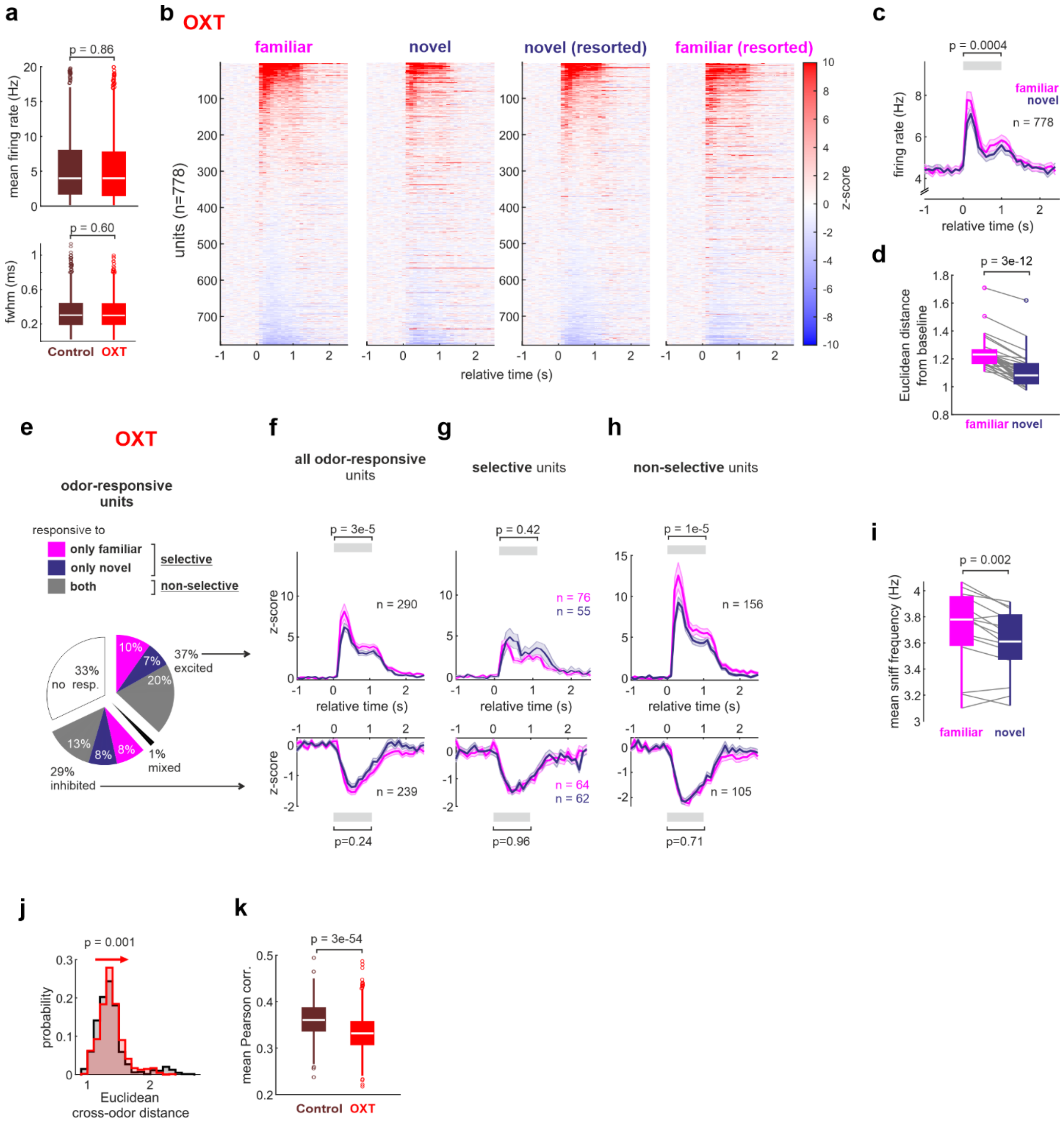

Supplementary Fig. 13, related to Figs. 7 and 8. Oxytocin-enabled familiarity memory traces in the AON. a Distributions of (top) average firing rate and (bottom) waveform width (fwhm) of the AON units in the boosted OXT condition. **b** Trial-averaged z-scored response for each recorded unit to the different odor types: familiar and novel conspecifics. Units in the left two panels are sorted based on their mean response to the familiar odor during 0 to +1 s relative to odor onset. Responses in the right two panels are resorted according to the novel odor unit activity again during 0 to +1 s relative to odor onset. **c** Temporal evolution of the mean firing rate across all recorded units (mean ± SEM). Two-sided paired t-test comparing the mean firing rate, per unit, for familiar and novel odor stimuli were conducted for the 1 s odor duration window (number of units indicated). **d** The Euclidean distance from baseline was compared for the responses to familiar and novel emitters (0 to +1 s relative to odor onset); indicating a significantly stronger population response to the familiar odor (two-sided paired t-test; same as in Fig. 7c, but visualized with individual values for familiar and novel). **e** Neuronal response tuning of recorded units. For every odor type, the trial-averaged firing rate response was tested against baseline (two-sided Wilcoxon signed-rank test with Bonferroni correction for multiple comparisons). Significant increases in firing were termed odor-excited responses and significant decreases in spike rate were termed odor-inhibited responses. Mixed responses refer to units that show both significant odor-excited and odor-inhibited bins in the tested odor presentation period. Pie charts show fractions of units

responding to one or more of the odors. **f** The temporal evolution of mean z-scored rate responses of (top) odor-excited and (bottom) odor-inhibited units (mean ± SEM). Units were considered odor-excited if activated by at least one of the two odors and vice versa for odor-inhibited cells (two-sided Wilcoxon signed rank-test comparing the mean z-scored response per unit. The corresponding p-values for the 1 s odor presentation window shown together with the respective line plots. **g** Similar to f, but for “selective” units that were significantly activated by either the familiar or the novel odor (two-sided Wilcoxon rank sum test was used to compare the mean z-score response per unit. The corresponding p-values for the 1 s odor presentation window are shown together with the respective line plots). **h** Similar to f, but for “non-selective” units that were significantly activated by both the familiar and novel odor. **i** The trial-averaged sniff frequency responses to the smell of the familiar and novel animal shows higher sniff frequencies in response to the familiar one also in sessions with OXT release during the initial interaction (n = 15 mice with 1 session each; two-sided paired t-test for session averages). **j** Same as Fig. 8b, but with the first 5 trials of the session included (n = 900 trial combinations). **k** The mean Pearson correlation coefficients of the population vectors between familiar and novel odor responses (all cross-odor trial combinations and averaged from 0 to +1 s relative to odor onset) were compared using a two-sided Wilcoxon rank sum test (n = 900 trial combinations) and suggest less overlap in population response coding after OXT boost during initial exploration.

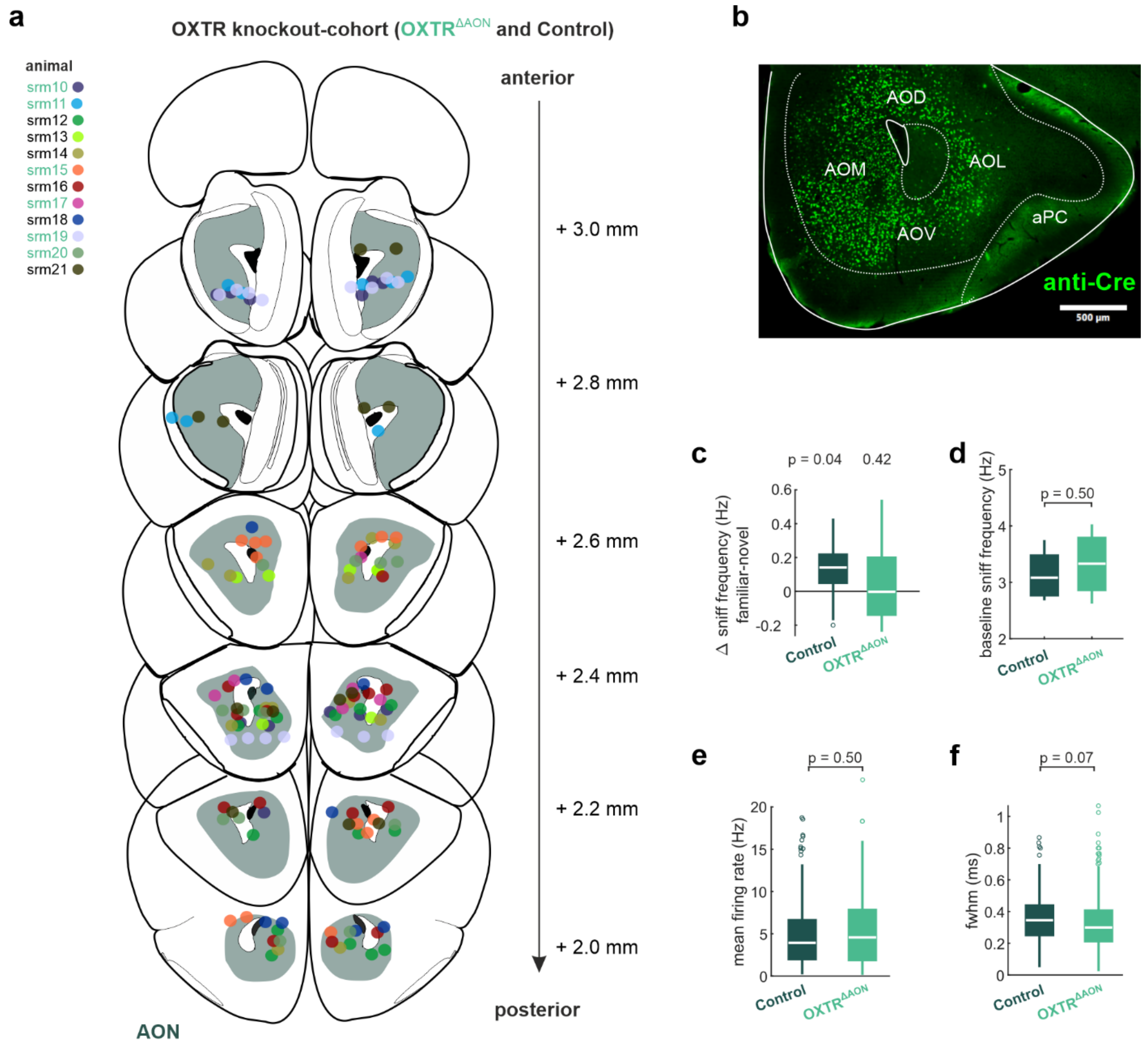

Supplementary Fig. 14, related to Figs. 7 and 8. OXT in the AON is required for the formation of familiarity memory traces. a Histological confirmation of recording sites for tetrode recordings in the AON (6 OXTR$^{\Delta AON}$ mice in cyan and 6 matched control OXTR$_{fl/fl}$ mice in black). Tetrode tip locations mapped onto the Allen Reference Atlas – Mouse Brain with coordinates indicated relative to bregma. Animal identities are color-coded. The anatomical contours were drawn based on the Allen Reference Atlas – Mouse Brain, available from atlas.brain-map.org[1]. **b** Example of expression pattern of AAV-delivered Cre in an OXTR$^{fl/fl}$ mouse in the AON. Contours were drawn based on the Allen Reference Atlas – Mouse Brain, available from atlas.brain-map.org[1]. Viral expression is restricted to the subdivisions of the AON. **c** The trial-averaged sniff frequency responses to the smell of the familiar and novel animals show higher sniff frequencies in response to the familiar one in the control group, but loss of differentiation in the OXTR$^{\Delta AON}$ group (n = 6 mice with 2 session each in the control and OXTR$^{\Delta AON}$ groups; two-sided paired t-test for within-group comparison between familiar and novel odor). **d** Baseline sniff frequencies were compared between the control and OXTR$^{\Delta AON}$ groups and show no differences (two-sided two-sample t-test, same data as in d) **e-f** Distributions of (e) average firing rate and (f) waveform width (fwhm) of the AON units in the control and OXTR$^{\Delta AON}$ groups (n = 275 units in the control and n = 164 units in the OXTR$^{\Delta AON}$ groups).

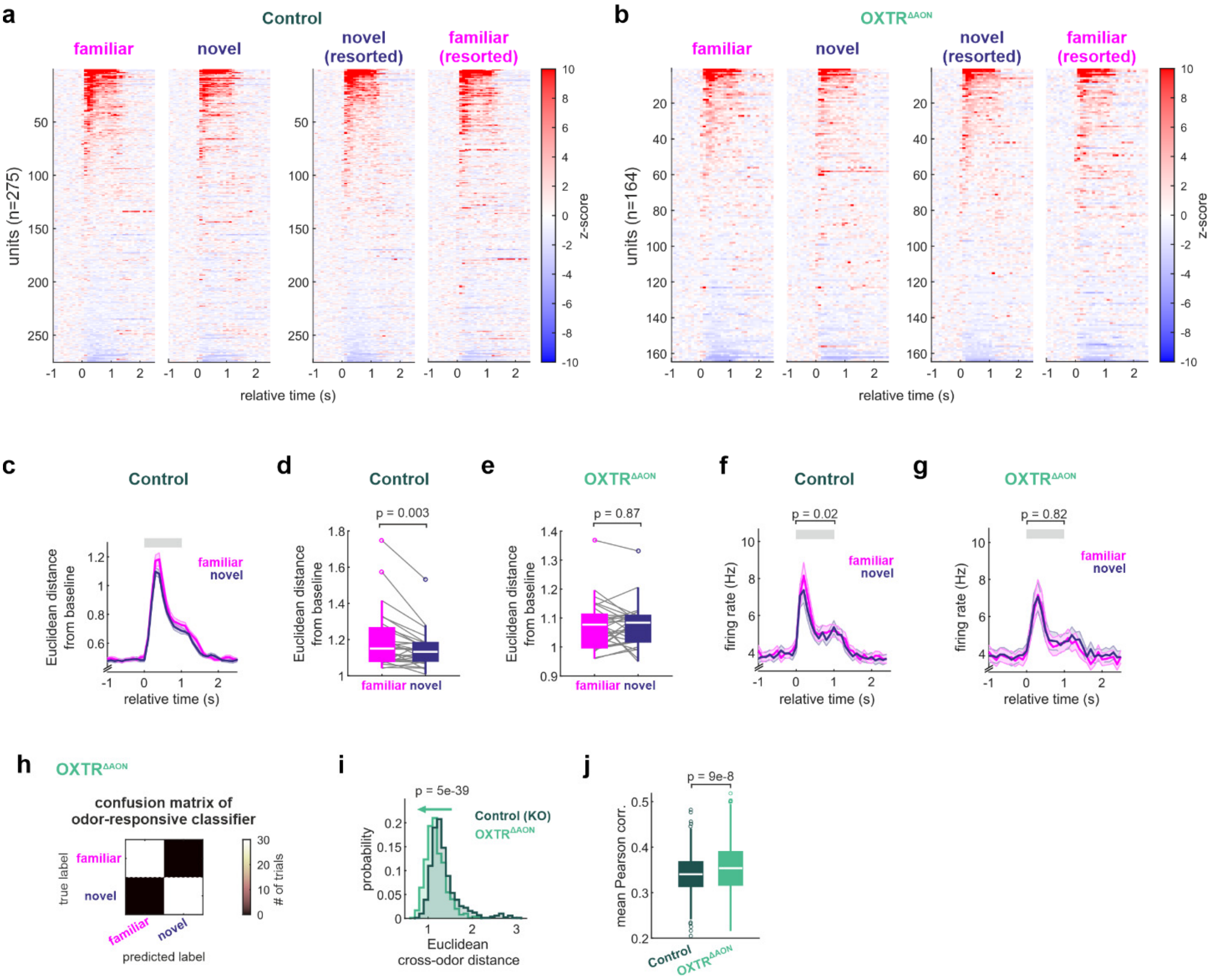

Supplementary Fig. 15, related to Figs. 7 and 8. OXT in the AON is required for the formation of familiarity memory traces. a-b Trial-averaged z-scored response for each recorded unit to the different odor types: familiar and novel conspecifics in the control group of the (a) Control and (b) OXTR$^{\Delta AON}$ groups. Units in the left two panels are sorted based on their mean response to the familiar odor during 0 to +1 s relative to odor onset. Responses in the right two panels are resorted according to the novel odor unit activity again during 0 to +1 s relative to odor onset. **c** The temporal evolution of the Euclidean distance from baseline of the population vectors from the Control group (mean ± SEM, n = 30 trials per odor). **d-e** The comparison of the mean Euclidean distance from baseline for the responses to familiar and novel emitters (0 to +1 s relative to odor onset); indicating a significantly stronger population response to the familiar odor in the (d) Control group but not in the (e) OXTR$^{\Delta AON}$ group (two-sided paired t-test, n = 30 trials per odor; same as in Fig. 7f but visualized with individual values for familiar and novel). **f-g** The temporal evolution of mean firing rate across all recorded units (mean ± SEM) in the (f) Control (n = 275 units) and (g) OXTR$^{\Delta AON}$ groups (n = 164 units). Two-sided paired t-test comparing the mean firing rate, per unit, for familiar and novel odor stimuli were conducted in the 1 s odor duration window. **h** The confusion matrix of a linear decoder, which was trained to predict the odor identity of a single trial from the neuronal population activity in OXTR$^{\Delta AON}$ animals, shows high accuracy, consistent with intact identity coding. Prediction accuracy was determined on trials, that were not included in the training dataset. A two-sided fisher's exact test against decoder models trained with shuffled labels indicate prediction accuracy significantly above chance level (p = 1e-14). **i** Same as Fig. 8c, but with the first 5 trials of the session included (n = 900 trial combinations). **j** The mean Pearson correlation coefficients of the population vectors between familiar and novel odor responses (all cross-odor trial combinations and averaged from 0 to +1 s relative to odor onset) were compared using a two-sided Wilcoxon rank sum test) and suggest more overlap in population response coding in the OXTR$^{\Delta AON}$ group.

Supplementary Table 1, related to Fig. 1. Number of emitter mice used in the different cohorts.

This table contains the number of unique emitter mice used in the different experiments. Note that different recording condition groups were run with partially overlapping emitter animals. This pool of animals was used to draw different combinations for every session, avoiding the repetition of unique combinations.

	number of receiver animals	size of C57BL/6 #1 emitter pool	size of C57BL/6 #2 emitter pool	size of CD1 emitter pool
AON	8	4	3	2
pPC/LEC	11	5	4	4
MOB	8	3	3	2
VTA	3	3	3	3

Supplementary Table 2, related to Figs. 2-8. Number of emitter mice used in the different cohorts.

This table contains the number of unique emitter mice used in the different experiments. Note that different recording condition groups were run with partially overlapping emitter animals. This pool of animals was used to draw different combinations for every session, avoiding the repetition of unique combinations. The same emitter served as familiar animal in a session with one receiver and as novel animal in another session with a different receiver.

	number of receiver animals	size of C57BL/6 emitter pool
AON $ChR2^{OXT/PVN}$	8	15
pPC/LEC	11	5
VTA	3	5
MOB	8	15
photometry	9	10
$OXTR^{\Delta AON}$	12	10

Supplementary Table 3, related to Fig. 5. Oxytocin modulates network activity in awake mice.

This table contains the SPM results table corresponding to Fig. 5c (two-sided t-test and Family wise error cluster-correction (FWEc) with a cluster-defining threshold of pCDT < 0.01 and pFWEc < 0.05). The cluster with k_E = 463 corresponds to the posterior cluster and the one with k_E = 115 corresponds to the anterior cluster.

set-level		cluster-level				peak-level					mm mm mm
p	c	$p_{FWE\text{-}corr}$	$q_{FDR\text{-}corr}$	k_E	p_{uncorr}	$p_{FWE\text{-}corr}$	$q_{FDR\text{-}corr}$	T	$(Z_{\equiv})$	p_{uncorr}	
0.000	2	0.000		463	0.000	0.003	0.003	6.75	4.92	0.000	-6 -15 -48
						0.166	0.035	4.86	3.96	0.000	-39 -30 -24
						0.185	0.035	4.79	3.93	0.000	6 -18 -48
		0.012		115	0.001	0.094	0.032	5.18	4.15	0.000	-18 24 -36
						0.698	0.047	3.82	3.31	0.000	-9 18 -36
						0.811	0.052	3.63	3.18	0.001	6 18 -42

Height threshold: T = 2.51, p = 0.010 (1.000)
Extent threshold: k = 115 voxels, p = 0.001 (0.012)
Expected voxels per cluster, <k> = 9.060
Expected number of clusters, <c> = 0.01
FWEp: 5.458, FDRp: 3.739, FWEc: 115

Degrees of freedom = [1.0, 22.0]
FWHM = 13.4 10.4 14.7 mm mm mm; 4.5 3.5 2.5 {voxels}
Volume: 308124 = 5706 voxels = 113.2 resels
Voxel size: 3.0 3.0 6.0 mm mm mm; (resel = 37.81 voxels)

Supplementary Table 4, related to Figs. 1-8. Statistical tests.

Description and results of the statistical tests displayed in the manuscript figures. The first column of the table indicates the figure number and panel of the corresponding figure.

<table>
<tr><th>Figure</th><th>Sample Size</th><th>Statistical Test</th><th>Values</th></tr>
<tr><td>1e, AON</td><td><table><tr><td></td><td>Correct prediction</td><td>Incorrect prediction</td></tr><tr><td>real</td><td>100</td><td>0</td></tr><tr><td>shuffle</td><td>28</td><td>72</td></tr></table></td><td>Two-sided Fisher’s exact test</td><td>p = 3e-31</td></tr>
<tr><td>1e, pPC</td><td><table><tr><td></td><td>Correct prediction</td><td>Incorrect prediction</td></tr><tr><td>real</td><td>92</td><td>8</td></tr><tr><td>shuffle</td><td>21</td><td>79</td></tr></table></td><td>Two-sided Fisher’s exact test</td><td>p = 5e-26</td></tr>
<tr><td>1e, LEC</td><td><table><tr><td></td><td>Correct prediction</td><td>Incorrect prediction</td></tr><tr><td>real</td><td>95</td><td>5</td></tr><tr><td>shuffle</td><td>21</td><td>79</td></tr></table></td><td>Two-sided Fisher’s exact test</td><td>p = 4e-29</td></tr>
<tr><td>1g, AON</td><td>[20, 20, 20, 20, 20]</td><td>Repeated measures one-way ANOVA with Geisser-Greenhouse correction and two-sided Tukey’s post-hoc test for multiple comparisons</td><td>0 to +1 s relative to odor onset:
F (2.777, 52.77) = 30.17, p < 0.0001; post-hoc comparisons: p(C57BL/6#1 vs. C57BL/6#2) = 0.86, p(C57BL/6#1 vs. CD1) = 0.0009, p(C57BL/6#1 vs. ylang ylang) < 0.0001, p(C57BL/6#1 vs. peanut butter) = 0.99, p(C57BL/6#2 vs. CD1) = 0.0012, p(C57BL/6#2 vs. ylang ylang) < 0.0001, p(C57BL/6#2 vs. peanut butter) = 0.87, p(CD1 vs. ylang ylang) = 0.03, p(CD1 vs. peanut butter) = 0.0005, p(ylang ylang vs. peanut butter) < 0.0001</td></tr>
<tr><td>1g, pPC</td><td>[20, 20, 20, 20, 20]</td><td>Repeated measures one-way ANOVA with Geisser-Greenhouse correction and two-sided Tukey’s post-hoc test for multiple comparisons</td><td>0 to +1 s relative to odor onset:
F (3.411, 64.81) = 18.61, p < 0.0001; post-hoc comparisons: p(C57BL/6#1 vs. C57BL/6#2) = 0.83, p(C57BL/6#1 vs. CD1) < 0.0001, p(C57BL/6#1 vs. ylang ylang) = 0.002, p(C57BL/6#1 vs. peanut butter) < 0.0001, p(C57BL/6#2 vs. CD1) = 0.0008, p(C57BL/6#2 vs. ylang ylang) = 0.003, p(C57BL/6#2 vs. peanut butter) = 0.0004, p(CD1 vs. ylang ylang) = 0.98, p(CD1 vs. peanut butter) = 0.67, p(ylang ylang vs. peanut butter) = 0.51</td></tr>
<tr><td>1g, LEC</td><td>[20, 20, 20, 20, 20]</td><td>Repeated measures one-way ANOVA with Geisser-Greenhouse correction and two-sided Tukey’s post-hoc test for multiple comparisons</td><td>0 to +1 s relative to odor onset:
F (2.857, 54.29) = 1.970, p = 0.13; post-hoc comparisons: p(C57BL/6#1 vs. C57BL/6#2) = 0.72, p(C57BL/6#1 vs. CD1) = 0.50, p(C57BL/6#1 vs. ylang ylang) = 0.99, p(C57BL/6#1 vs. peanut butter) = 0.99, p(C57BL/6#2 vs. CD1) = 0.86, p(C57BL/6#2 vs. ylang ylang) = 0.87, p(C57BL/6#2 vs. peanut butter) = 0.24, p(CD1 vs. ylang ylang) = 0.45, p(CD1 vs. peanut butter) = 0.01, p(ylang ylang vs. peanut butter) = 0.97</td></tr>
<tr><td>1h</td><td>[13, 13, 13, 13, 13]</td><td>Repeated measures one-way</td><td>0 to +1 s relative to odor onset:
F (2.451, 29.41) = 6.700, p = 0.002; post-hoc comparisons: p(C57BL/6#1 vs. C57BL/6#2) = 0.24,</td></tr>
</table>

<table>
<tr><td></td><td></td><td>ANOVA with Geisser-Greenhouse correction and two-sided Tukey's post-hoc test for multiple comparisons</td><td>p(C57BL/6#1 vs. CD1) = 0.99, p(C57BL/6#1 vs. ylang ylang) = 0.11, p(C57BL/6#1 vs. peanut butter) = 0.005, p(C57BL/6#2 vs. CD1) = 0.73, p(C57BL/6#2 vs. ylang ylang) = 0.21, p(C57BL/6#2 vs. peanut butter) = 0.009, p(CD1 vs. ylang ylang) = 0.11, p(CD1 vs. peanut butter) = 0.049, p(ylang ylang vs. peanut butter) = 0.99</td></tr>
<tr><td>S4e, MOB</td><td><table><tr><td></td><td>Correct prediction</td><td>Incorrect prediction</td></tr><tr><td>real</td><td>93</td><td>7</td></tr><tr><td>shuffle</td><td>13</td><td>87</td></tr></table></td><td>Two-sided Fisher's exact test</td><td>p = 4e-33</td></tr>
<tr><td>S4e, VTA</td><td><table><tr><td></td><td>Correct prediction</td><td>Incorrect prediction</td></tr><tr><td>real</td><td>84</td><td>16</td></tr><tr><td>shuffle</td><td>22</td><td>78</td></tr></table></td><td>Two-sided Fisher's exact test</td><td>p = 3e-19</td></tr>
<tr><td>S4g, MOB</td><td>[20, 20, 20, 20, 20]</td><td>Repeated measures one-way ANOVA with Geisser-Greenhouse correction and two-sided Tukey's post-hoc test for multiple comparisons</td><td>0 to +1 s relative to odor onset:
F (1.816, 34.51) = 78.05, p < 0.0001; post-hoc comparisons: p(C57BL/6#1 vs. C57BL/6#2) = 0.74, p(C57BL/6#1 vs. CD1) = 0.0005, p(C57BL/6#1 vs. ylang ylang) < 0.0001, p(C57BL/6#1 vs. peanut butter) = 0.73, p(C57BL/6#2 vs. CD1) < 0.0001, p(C57BL/6#2 vs. ylang ylang) < 0.0001, p(C57BL/6#2 vs. peanut butter) = 0.15, p(CD1 vs. ylang ylang) < 0.0001, p(CD1 vs. peanut butter) = 0.007, p(ylang ylang vs. peanut butter) < 0.0001</td></tr>
<tr><td>S4j, VTA</td><td>[20, 20, 20, 20, 20]</td><td>Repeated measures one-way ANOVA with Geisser-Greenhouse correction and two-sided Tukey's post-hoc test for multiple comparisons</td><td>0 to +1 s relative to odor onset:
F (3.039, 57.73) = 10.06, p < 0.0001; post-hoc comparisons: p(C57BL/6#1 vs. C57BL/6#2) = 0.99, p(C57BL/6#1 vs. CD1) = 0.99, p(C57BL/6#1 vs. ylang ylang) = 0.09, p(C57BL/6#1 vs. peanut butter) = 0.04, p(C57BL/6#2 vs. CD1) = 0.99, p(C57BL/6#2 vs. ylang ylang) = 0.02, p(C57BL/6#2 vs. peanut butter) = 0.001, p(CD1 vs. ylang ylang) = 0.45, p(CD1 vs. peanut butter) = 0.01, p(ylang ylang vs. peanut butter) < 0.0001</td></tr>
<tr><td rowspan="5">S5b, 0-0.33 s</td><td rowspan="5">[13,13]; from 13 animals</td><td>two-tailed paired t-test (C57BL/6#1 vs. baseline)</td><td>t(12) = 5.0, p = 0.0003</td></tr>
<tr><td>two-tailed paired t-test (C57BL/6#2 vs. baseline)</td><td>t(12) = 5.7, p = 9e-5</td></tr>
<tr><td>two-tailed paired t-test (CD1 vs. baseline)</td><td>t(12) = 5.7, p = 0.0001</td></tr>
<tr><td>two-tailed paired t-test (ylang ylang vs. baseline)</td><td>t(12) = 4.3, p = 0.001</td></tr>
<tr><td>two-tailed paired t-test (peanut</td><td>t(12) = 5.5, p = 0.0001</td></tr>
</table>

		butter vs. baseline)	
S5b, 0-1 s	[13,13]; from 13 animals	two-tailed paired t-test (C57BL/6#1 vs. baseline)	**t(12) = 3.8, p = 0.0027**
		two-tailed paired t-test (C57BL/6#2 vs. baseline)	**t(12) = 3.8, p = 0.0023**
		two-tailed paired t-test (CD1 vs. baseline)	**t(12) = 2.7, p = 0.0193**
		two-tailed paired t-test (ylang ylang vs. baseline)	t(12) = -0.6, p = 0.56
		two-tailed paired t-test (peanut butter vs. baseline)	t(12) = -1.1, p = 0.31
S5c, 0-0.33s	[13, 13, 13, 13, 13]	Repeated measures one-way ANOVA with Geisser-Greenhouse correction and two-sided Tukey's post-hoc test for multiple comparisons	F (3.156, 37.87) = 2.102, p = 0.11
S5c, 0-1s	See Fig. 1h		
S5f	[13, 13, 13, 13, 13]	Repeated measures one-way ANOVA with Geisser-Greenhouse correction and two-sided Tukey's post-hoc test for multiple comparisons	**F (2.583, 30.99) = 14.41, p < 0.0001**; post-hoc comparisons: p(C57BL/6#1 vs. C57BL/6#2) = 0.99, p(C57BL/6#1 vs. CD1) = 0.71, **p(C57BL/6#1 vs. ylang ylang) = 0.01**, p(C57BL/6#1 vs. peanut butter) = 0.92, p(C57BL/6#2 vs. CD1) = 0.49, **p(C57BL/6#2 vs. ylang ylang) = 0.0006**, p(C57BL/6#2 vs. peanut butter) = 0.97, **p(CD1 vs. ylang ylang) = 0.0009**, p(CD1 vs. peanut butter) = 0.22, **p(ylang ylang vs. peanut butter) < 0.0001**
2b	[15, 15]	two-tailed paired t-test	**t(14) = 3.93, p = 0.002**
2c	[30, 30]	two-tailed paired t-test	**t(29) = 6.5, p = 4e-7**
2e	[30, 30]	two-tailed paired t-test	**t(29) = 5.7, p = 4e-6**
2f	[30, 30]	two-tailed paired t-test	t(29) = 0.9, p = 0.40
2g	[30, 30]	two-tailed paired t-test	**t(29) = 3.0, p = 0.0054**
S6c	[38, 38]	two-tailed paired t-test	**t(37) = 2.1, p = 0.04**
S8a, AON	[769, 769]	two-tailed paired t-test	0.0 – 1.0s window: **t(768) = 3.1, p = 0.002**
S8a, pPC	[127, 127]	two-tailed paired t-test	0.0 – 1.0s window**: t(126) = 3.2, p = 0.0020**

S8a, LEC	[83, 83]	two-tailed paired t-test	0.0 – 1.0s window: t(82) = 0.4, p = 0.67
S8a, VTA	[46, 46]	two-tailed paired t-test	0.0 – 1.0s window: **t(45) = 2.5, p = 0.0155**
S8c, AON (odor-excited)	[296, 296]	two-sided Wilcoxon signed rank test (familiar – novel)	0.0 – 1.0s window: **Z = 3.9, p = 9e-5**
S8c, AON (odor-inhibited)	[249, 249]	two-sided Wilcoxon signed rank test (familiar – novel)	0.0 – 1.0s window: Z = -0.5, p = 0.64
S8c, pPC (odor-excited)	[28, 28]	two-sided Wilcoxon signed rank test (familiar – novel)	0.0 – 1.0s window: **Z = 2.0, p = 0.04**
S8c, pPC (odor-inhibited)	[43, 43]	two-sided Wilcoxon signed rank test (familiar – novel)	0.0 – 1.0s window: Z = 1.1, p = 0.27
S8c, LEC (odor-inhibited)	[16, 16]	two-sided Wilcoxon signed rank test (familiar – novel)	0.0 – 1.0s window: Z = 0.6, p = 0.57
S8c, LEC (odor-inhibited)	[27, 27]	two-sided Wilcoxon signed rank test (familiar – novel)	0.0 – 1.0s window: Z = -0.7, p = 0.50
S8c, VTA (odor-excited)	[13, 13]	two-sided Wilcoxon signed rank test (familiar – novel)	0.0 – 1.0s window: **p = 0.04**
S8c, VTA (odor-inhibited)	[5, 5]	two-sided Wilcoxon signed rank test (familiar – novel)	0.0 – 1.0s window: p = 0.44
S8d, AON (odor-excited)	[71, 46]	two-sided Wilcoxon rank sum test (familiar vs. novel)	0.0 – 1.0s window: Z = 1.9, p = 0.06
S8d, AON (odor-inhibited)	[60, 63]	two-sided Wilcoxon rank sum test (familiar vs. novel)	0.0 – 1.0s window: Z = -1.0, p = 0.33
S8e, AON (odor-excited)	[175, 175]	two-sided Wilcoxon signed rank test (familiar – novel)	0.0 – 1.0s window: **Z = 2.7, p = 0.007**
S8e, AON (odor-inhibited)	[123, 123]	two-sided Wilcoxon signed rank test (familiar – novel)	0.0 – 1.0s window: Z = -1.8, p = 0.08
3c, MOB	[23, 23]	two-tailed paired t-test (familiar vs. novel)	**t(22) = 3.6, p = 0.002**
3c, AON	[23, 23]	two-tailed paired t-test	**t(22) = 3.4, p = 0.002**

		(familiar vs. novel)	
3d	[750, 750]	two-sided Wilcoxon signed rank test	**Z = 2.3, p = 0.02**
3e	[23, 23]	two-sided Wilcoxon signed rank test	**Z = 2.7, p = 0.007**
4d	[18, 18]	Two-tailed paired t-test	**t(17) = 2.7, p = 0.020**
4g	[30, 30]	two-tailed paired t-test	**t(29) = 3.6, p = 0.0012**
S10b, MOB	[23, 23]	two-tailed paired t-test (familiar vs. novel)	**t(22) = 3.3, p = 0.004**
S10b, AON	[23, 23]	two-tailed paired t-test (familiar vs. novel)	**t(22) = 3.4, p = 0.003**
S10c	[23, 23]	two-sided Wilcoxon signed rank test	Z = 1.7, p = 0.09
S10e, AON	[23,23]	two-sided Wilcoxon signed rank test (mean latency per session: familiar - novel)	**Z = 2.4, p = 0.018**
S10e, MOB	[23,23]	two-sided Wilcoxon signed rank test (mean latency per session: familiar - novel)	**Z = 2.4, p = 0.016**
S11e	[49, 49] units	two-sided Wilcoxon signed rank test (firing rate per unit: familiar - novel)	0.0 – 1.0s window: Z = 0.5, p = 0.62
S11g	[30, 30]	two-tailed paired t-test	**t(29) = 3.4, p = 0.0021**
S11i, familiar	[15, 250] odor-excited units from MOB and AON respectively	two-sided Wilcoxon rank sum test	**Z = 2.5, p = 0.01**
S11i, novel	[12, 224] odor-excited units from MOB and AON respectively	two-sided Wilcoxon rank sum test	**Z = 3.3, p = 0.001**
S11j, familiar	[30, 30]	two-sided Wilcoxon rank sum test	**Z = 5.0, p = 5e-7**
S11j, novel	[30, 30]	two-sided Wilcoxon rank sum test	**Z = 3.9, p = 0.0001**
6b	<table><tr><td></td><td>Resp.</td><td>no resp.</td></tr><tr><td>block</td><td>10</td><td>364</td></tr></table>	two-sided Fisher's exact test	**p = 6e-8**

<table>
<tr><td></td><td><table><tr><td>OXT</td><td>80</td><td>599</td></tr></table></td><td></td><td></td></tr>
<tr><td></td><td><table><tr><td></td><td>Resp.</td><td>no resp.</td></tr><tr><td>heat</td><td>13</td><td>313</td></tr><tr><td>OXT</td><td>80</td><td>599</td></tr></table></td><td>two-sided Fisher’s exact test</td><td>p = 3e-5</td></tr>
<tr><td></td><td><table><tr><td></td><td>Resp.</td><td>no resp.</td></tr><tr><td>Block</td><td>10</td><td>364</td></tr><tr><td>heat</td><td>80</td><td>599</td></tr></table></td><td>two-sided Fisher’s exact test</td><td>p = 0.40</td></tr>
<tr><td>6b, repeated for laser-excited responses</td><td><table><tr><td></td><td>Exc.</td><td>no resp.</td></tr><tr><td>block</td><td>7</td><td>367</td></tr><tr><td>OXT</td><td>57</td><td>622</td></tr></table></td><td>two-sided Fisher’s exact test</td><td>p = 6e-6</td></tr>
<tr><td></td><td><table><tr><td></td><td>Exc.</td><td>no resp.</td></tr><tr><td>heat</td><td>13</td><td>313</td></tr><tr><td>OXT</td><td>57</td><td>622</td></tr></table></td><td>two-sided Fisher’s exact test</td><td>p = 0.01</td></tr>
<tr><td></td><td><table><tr><td></td><td>Exc.</td><td>no resp.</td></tr><tr><td>block</td><td>7</td><td>367</td></tr><tr><td>heat</td><td>13</td><td>313</td></tr></table></td><td>two-sided Fisher’s exact test</td><td>p = 0.11</td></tr>
<tr><td>6d</td><td>[28, 12, 14] for OXT, blocked control and heat control respectively</td><td>Kruskal-Wallis test with post-hoc two-sided Tukey’s honest significance test for multiple comparisons</td><td>F(2,51) = 20.4, p = 3e-7; post-hoc comparisons: p(OXT vs. blocked) = 5e-5, p(OXT vs. heat) = 3e-6, p(blocked vs heat) = 0.9</td></tr>
<tr><td>7c, control</td><td colspan="3">See Fig. 2c (AON)</td></tr>
<tr><td>7c, OXT</td><td>[30, 30]</td><td>two-tailed paired t-test</td><td>t(29) = 11.4, p = 3e-12</td></tr>
<tr><td>7c, OXT vs. control</td><td>[30, 30]</td><td>two-tailed two-sample t-test</td><td>t(58) = -4.5, p = 3.8e-5</td></tr>
<tr><td>7f, control</td><td>[30, 30]</td><td>two-tailed paired t-test</td><td>t(29) = 3.3, p = 0.003</td></tr>
<tr><td>7f, $OXTR^{\Delta AON}$</td><td>[30, 30]</td><td>two-tailed paired t-test</td><td>t(29) = 0.2, p = 0.87</td></tr>
<tr><td>7f, control versus $OXTR^{\Delta AON}$</td><td>[30, 30]</td><td>two-tailed two-sample t-test</td><td>t(58) = 2.4, p = 0.002</td></tr>
<tr><td>Related to 8a</td><td>500 iterations per subsample size</td><td>Likelihood Ratio Test to test if including “number of subsampled units” improves model fit</td><td>$X^2(10)$ = 6.3, p = 0.79</td></tr>
<tr><td>8a</td><td>500 iterations per subsample size</td><td>two-sided Tukey’s post-hoc test with Bonferroni adjustment for linear mixed-effects model.</td><td>Control (KO) – Control (OXT): t = -7.0, p = 2e-12
OXT – Control (OXT): t = 34, p = 2e-16
$OXTR^{\Delta AON}$ – Control (OXT): t = -34, p = 2e-16
OXT – Control (KO): t = 41, p = 2e-16
$OXTR^{\Delta AON}$ – Control (KO): t = -27, p = 2e-16
$OXTR^{\Delta AON}$ – OXT: t = -68, p = 2e-16</td></tr>
</table>

8b	[625, 625]	two-sided Wilcoxon rank sum test	**Z = -4.6, p = 5e-6.**<br>median(Control) = 1.29<br>median(OXT) = 1.32
8c	[625, 625]	two-sided Wilcoxon rank sum test	**Z = 11.2, p = 4e-29.**<br>median(Control) = 1.21<br>median(KO) = 1.10
S13a, mean firing rate	[769, 778]	Two-tailed two-sample t-test	t(1545) = 0.18
S13a, fwhm	[769, 778]	Two-tailed two-sample t-test	t(1535) = 0.53
S13c	[778, 778]	Two-tailed paired t-test	0.0 – 1.0s window: **t(777) = 3.5, p = 0.0004**
S13f (odor-excited)	[290, 290]	two-sided Wilcoxon signed rank test	0.0 – 1.0s window: **Z = 4.2, p = 3e-5**
S13f (odor-inhibited)	[239, 239]	two-sided Wilcoxon signed rank test	0.0 – 1.0s window: Z = -1.2, p = 0.24
S13g (odor-excited)	[76, 55]	two-sided Wilcoxon rank sum test (familiar vs. novel)	0.0 – 1.0s window: Z = -0.8, p = 0.42
S13g (odor-inhibited)	[64, 62]	two-sided Wilcoxon rank sum test (familiar vs. novel)	0.0 – 1.0s window: Z = 0.05, p = 0.96
S13h (odor-excited)	[156, 156]	two-sided Wilcoxon signed rank test (familiar – novel)	0.0 – 1.0s window: **Z = 4.4, p = 1e-5**
S13h (odor-inhibited)	[105, 105]	two-sided Wilcoxon signed rank test (familiar – novel)	0.0 – 1.0s window: Z = -0.4, p = 0.71
S13i	[15,15]	two-tailed one sample t-test	**t(14) = 3.90, p = 0.002**
S13j	[900, 900]	two-sided Wilcoxon rank sum test	**Z = -3.2, p = 0.0012**<br>median(Control) = 1.33<br>median(OXT) = 1.36
S13k	[900, 900]	two-sided Wilcoxon rank sum test	**Z = 15.5, p = 3e-54**
S14c, control (familiar vs. novel)	[12, 12]	two-tailed paired t-test	**t(11) = 2.2, p = 0.049**
S14c, $OXTR^{\Delta AON}$ (familiar vs. novel)	[12, 12]	two-tailed one sample t-test	t(11) = 0.8, p = 0.42
S14d	[12, 12]	two-tailed two-sample t-test	t(22) = -1.0, p = 0.50
S14e	[164, 275]	two-tailed two-sample t-test	t(437) = -0.7, p = 0.50

S14f	[164, 275]	two-tailed two-sample t-test	t(435) = 1.8, p = 0.07
S15f	[275, 275]	Two-tailed paired t-test	0.0 – 1.0s window: **t(274) = 2.4, p = 0.018**
S15g	[164, 164]	Two-tailed paired t-test	0.0 – 1.0s window: t(163) = -0.23, p = 0.82
S15i	[900, 900]	two-sided Wilcoxon rank sum test	**Z = 13.1, p = 5e-39**<br>median(control) = 1.27<br>median($OXTR^{KO}$) = 1.15
S15j	[900, 900]	two-sided Wilcoxon rank sum test	**Z = -5.3, p = 9e-8**

Supplementary References

1. Allen Reference Atlas – Mouse Brain [Adult Mouse]. Available from atlas.brain-map.org.
2. Sack, M. et al. Interactive tool to create adjustable anatomical atlases for mouse brain imaging. Magn. Reson. Mater. Phys. Biol. Med. 34, 183–187 (2021). Sack, M. *et al.* Interactive tool to create adjustable anatomical atlases for mouse brain imaging. *Magn. Reson. Mater. Phys. Biol. Med.* **34**, 183–187 (2021).
