# Supplementary material for: Oxytocin induces the formation of distinctive cortical representations and cognitions biased toward familiar mice
Source: Nat Commun. 2024 Jul 25;15:6274. doi: 10.1038/s41467-024-50113-6 (PMC11272796; doi:10.1038/s41467-024-50113-6)
Supplement: Supplementary file 3 — Reporting Summary [file 41467_2024_50113_MOESM3_ESM.pdf]

nature portfolio

Corresponding author(s): Wolfgang Kelsch

Last updated by author(s): May 25, 2024

Reporting Summary

Nature Portfolio wishes to improve the reproducibility of the work that we publish. This form provides structure for consistency and transparency in reporting. For further information on Nature Portfolio policies, see our Editorial Policies and the Editorial Policy Checklist.

Statistics

For all statistical analyses, confirm that the following items are present in the figure legend, table legend, main text, or Methods section.

n/a	Confirmed	
☐	☒	The exact sample size (*n*) for each experimental group/condition, given as a discrete number and unit of measurement
☐	☒	A statement on whether measurements were taken from distinct samples or whether the same sample was measured repeatedly
☐	☒	The statistical test(s) used AND whether they are one- or two-sided<br>*Only common tests should be described solely by name; describe more complex techniques in the Methods section.*
☐	☒	A description of all covariates tested
☐	☒	A description of any assumptions or corrections, such as tests of normality and adjustment for multiple comparisons
☐	☒	A full description of the statistical parameters including central tendency (e.g. means) or other basic estimates (e.g. regression coefficient) AND variation (e.g. standard deviation) or associated estimates of uncertainty (e.g. confidence intervals)
☐	☒	For null hypothesis testing, the test statistic (e.g. *F*, *t*, *r*) with confidence intervals, effect sizes, degrees of freedom and *P* value noted<br>*Give P values as exact values whenever suitable.*
☒	☐	For Bayesian analysis, information on the choice of priors and Markov chain Monte Carlo settings
☐	☒	For hierarchical and complex designs, identification of the appropriate level for tests and full reporting of outcomes
☐	☒	Estimates of effect sizes (e.g. Cohen's *d*, Pearson's *r*), indicating how they were calculated

Our web collection on statistics for biologists contains articles on many of the points above.

Software and code

Policy information about availability of computer code

Data collection	- Matlab 2017b/2020/2021a<br>- Arduino Software 1.8.5<br>- Intan Technologies Acquisition Software 1.4.2<br>- Bruker Paravision Software (Version 6)<br>- DinoCapture 2.0<br>- VS200 ASW V3.2.1<br>- LAS AF
Data analysis	- Custom code: https://github.com/KelschLAB/OXT-Wolf<br>- Matlab (2017a/2021a/2023a) with Statistics and Machine Learning Toolbox (version 12.5) and Curve Fitting Toolbox (version 3.9)<br>- Kilosort2 with Phy2 (https://github.com/cortex-lab/phy)<br>- MLIB toolbox (Version 6)<br>- spikes toolbox (04/21)<br>- FieldTrip (20210505)<br>- IoSR Matlab Toolbox (https://github.com/IoSR-Surrey/MatlabToolbox)<br>- Andreas Trier Poulsen (2023). mseb(x,y,errBar,lineProps,transparent) (https://www.mathworks.com/matlabcentral/fileexchange/47950-mseb-x-y-errbar-lineprops-transparent), MATLAB Central File Exchange. Retrieved November 27, 2023.<br>- Statistical Parametric Mapping 12 (SPM12)<br>- MRIcroGL (v1.2)<br>- R-Studio (4.0.3)

- Prism 9.5.1
- CorelDraw 2021.5

For manuscripts utilizing custom algorithms or software that are central to the research but not yet described in published literature, software must be made available to editors and reviewers. We strongly encourage code deposition in a community repository (e.g. GitHub). See the Nature Portfolio guidelines for submitting code & software for further information.

Data

Policy information about availability of data

All manuscripts must include a data availability statement. This statement should provide the following information, where applicable:
- Accession codes, unique identifiers, or web links for publicly available datasets
- A description of any restrictions on data availability
- For clinical datasets or third party data, please ensure that the statement adheres to our policy

The electrophysiology and fMRI data generated in this study are under active use by the reporting laboratory; all data presented in this manuscript are available upon request from the Lead Contact. Processed AON single-unit data are available for download at https://doi.org/10.6084/m9.figshare.24637986.v1. Source data are provided with this paper.

Research involving human participants, their data, or biological material

Policy information about studies with human participants or human data. See also policy information about sex, gender (identity/presentation), and sexual orientation and race, ethnicity and racism.

Reporting on sex and gender	*Use the terms sex (biological attribute) and gender (shaped by social and cultural circumstances) carefully in order to avoid confusing both terms. Indicate if findings apply to only one sex or gender; describe whether sex and gender were considered in study design; whether sex and/or gender was determined based on self-reporting or assigned and methods used.<br>Provide in the source data disaggregated sex and gender data, where this information has been collected, and if consent has been obtained for sharing of individual-level data; provide overall numbers in this Reporting Summary. Please state if this information has not been collected.<br>Report sex- and gender-based analyses where performed, justify reasons for lack of sex- and gender-based analysis.*
Reporting on race, ethnicity, or other socially relevant groupings	*Please specify the socially constructed or socially relevant categorization variable(s) used in your manuscript and explain why they were used. Please note that such variables should not be used as proxies for other socially constructed/relevant variables (for example, race or ethnicity should not be used as a proxy for socioeconomic status).<br>Provide clear definitions of the relevant terms used, how they were provided (by the participants/respondents, the researchers, or third parties), and the method(s) used to classify people into the different categories (e.g. self-report, census or administrative data, social media data, etc.)<br>Please provide details about how you controlled for confounding variables in your analyses.*
Population characteristics	*Describe the covariate-relevant population characteristics of the human research participants (e.g. age, genotypic information, past and current diagnosis and treatment categories). If you filled out the behavioural & social sciences study design questions and have nothing to add here, write "See above."*
Recruitment	*Describe how participants were recruited. Outline any potential self-selection bias or other biases that may be present and how these are likely to impact results.*
Ethics oversight	*Identify the organization(s) that approved the study protocol.*

Note that full information on the approval of the study protocol must also be provided in the manuscript.

Field-specific reporting

Please select the one below that is the best fit for your research. If you are not sure, read the appropriate sections before making your selection.

☒ Life sciences ☐ Behavioural & social sciences ☐ Ecological, evolutionary & environmental sciences

For a reference copy of the document with all sections, see nature.com/documents/nr-reporting-summary-flat.pdf

Life sciences study design

All studies must disclose on these points even when the disclosure is negative.

Sample size	Sample sizes (number of single units within a single analyses and number of animals per group) were chosen according to previously used numbers in published awake recordings (eg. Oettl et al. (2020), Nat. Comm. 11(1):3460 and Winkelmeier et al. (2022), Nat. Comm. 13(1):3305) sufficient to detect medium or large size effects. Since the exact variability of the effects was unknown, sample size calculations were not reliably possible.
Data exclusions	Single-units were only included in the further analyses if their fraction of spikes violating the refractory period was less than 2 %. We further analyzed AON units with a mean firing rate between 0.1 and 20 Hz and MOB, LEC and pPC units with a mean firing rate above 0.1 Hz. In the VTA we analyzed single units with firing features of dopamine neurons (mean firing rate between 0.1 and 12 Hz). For the spike-field coupling

analysis in the AON, 19 out of the 769 units were excluded from this analysis, since no reliable ppc could be estimated because of their low firing rate (mean firing rate: 0.53 Hz).

Replication	The study examined a novel phenomenon and did not systematically generate full replication cohorts. However, the key finding of reinforced cortical representations of familiarity and higher salience assigned to the familiar odor were replicated in an independent cohort (matched control group for the OXT receptor knockout intervention).
Randomization	For the OXT receptor knockout experiment, subjects were randomized to either the intervention or control group. For the other cohorts, all mice received the same treatment, so no randomization was necessary at the subject level.<br>Trial sequences within a session were pseudo-randomized: No stimulus was consecutively applied more than three times in a row.<br>Further, the trial duration was randomly drawn from a uniform distribution between 10 and 12 s.
Blinding	Blinding was not relevant as all animals received the same treatment (paradigms and analyses).

Reporting for specific materials, systems and methods

We require information from authors about some types of materials, experimental systems and methods used in many studies. Here, indicate whether each material, system or method listed is relevant to your study. If you are not sure if a list item applies to your research, read the appropriate section before selecting a response.

Materials & experimental systems

n/a	Involved in the study	
☐	☒	Antibodies
☒	☐	Eukaryotic cell lines
☒	☐	Palaeontology and archaeology
☐	☒	Animals and other organisms
☒	☐	Clinical data
☒	☐	Dual use research of concern
☒	☐	Plants

Methods

n/a	Involved in the study	
☒	☐	ChIP-seq
☒	☐	Flow cytometry
☐	☒	MRI-based neuroimaging

Antibodies

Antibodies used	Primary: anti-oxytocin (PS38, mouse, kindly provided by Dr. Harold Gainer, NIH, Bethesda, USA), anti-Cre (rabbit, Millipore Cat# 69050-3, RRID:AB_10806983), anti-GFP (chicken, Abcam Cat# ab13970, RRID:AB_300798)<br>Secondary: Goat anti-Mouse, Alexa Fluor™ 488 (ThermoFisher Invitrogen, cat. n. A-11001, RRID:AB_2534069), Goat anti-Rabbit, Alexa Fluor™ 488 (ThermoFisher Invitrogen Cat# A-11008, RRID:AB_143165), Goat Anti-Chicken, Alexa Fluor 488 (ThermoFisher Invitrogen Cat# A-11039, RRID:AB_142924)
Validation	Validation of anti-oxytocin (PS38) immunohistochemistry in rat was first reported in (Ben-Barak et al., J. of Neurosci., 1985). Anti-OXT and anti-Cre antibodies were validated in the same application in Oettl et al., Neuron 2016. Anti-Cre, anti-GFP and secondary antibodies are comercially available and validated by the vendors.

Animals and other research organisms

Policy information about studies involving animals; ARRIVE guidelines recommended for reporting animal research, and Sex and Gender in Research

Laboratory animals	In the experiments, the following transgenic mice were used (at least 12 weeks old at the beginning of the experiment): 29 male heterozygous OXT-Cre mice (B6;129S-Oxttm1.1(cre)Dolsn/J, RRID:IMSR_JAX:024234, Jackson Laboratory) divided into 6 animals for histological confirmation and quantification of viral expression and 23 animals for neuroimaging, of which 16 proceeded to the in-vivo electrophysiology cohort, with 8 animals for recordings from the AON and 8 animals for recordings of multisite LFP and single-units from the MOB. Twelve male homozygous OXTRfl/fl mice (B6.129(SJL)-Oxtrtm1.1Wsy/J, RRID: IMSR_JAX:008471, obtained from W.S. Young, NIMH) were randomly assigned to the Cre injection or control group at a ratio of 1:1. All transgenic mice were bred in-house and maintained in a C57BL/6J (Charles River Laboratories) background (>F10). 3 male mice for in-vivo recording from VTA. 21 male C57BL/6J mice were obtained from Charles River Laboratories for in-vivo recording from LEC and pPC (n = 11) and for fiber photometry of top-down projections from the AON to the MOB (n = 10). We used a pool of emitter mice (Supplementary Table 1 and 2). Emitter mice were either male C57BL/6J mice or male CD1 mice from Charles River Laboratories.<br>The number of receiver and emitter mice in each experiment is given in Supplementary Tables 1 and 2.<br><br>All emitter mice were single housed in fresh cages for at least 24 hours before the experiment to prevent cross-contamination of odors from other cage mates. Animals were single housed following surgical procedures, supplied with ad-libitum access to food and water for the complete duration of the experiments and kept on a 12-hour light-dark-cycle (room temperature 24°C, air humidity 55%).<br><br>All procedures were approved by the local animal welfare authority (Regierungspräsidium Karlsruhe) and in accordance with the EU Directive 2010/63.

Wild animals	No wild animals were used in the study.
Reporting on sex	Only male mice were used in the study.
Field-collected samples	No field collected samples were used in the study.
Ethics oversight	Referat 35, Regierungspraesidium Karlsruhe, State of Baden-Wuerttemberg, Germany

Note that full information on the approval of the study protocol must also be provided in the manuscript.

Magnetic resonance imaging

Experimental design

Design type	event-related design
Design specifications	Each session had 4 trials of optogenetic stimulation with an inter-trial interval of 5 minutes. In each trial, the burst stimulation consisted of a train of 60 blue laser pulses at 30 Hz with a pulse duration of 5 ms.
Behavioral performance measures	No behavioral performance measures were acquired.

Acquisition

Imaging type(s)	Functional Magnetic Resonance Imaging
Field strength	9.4 Tesla (MRI scanner: 94/20 Bruker Biospec, Ettlingen Germany
Sequence & imaging parameters	Functional scans were acquired with a gradient-echo echo planar imaging (GE-EPI) sequence, with the following parameters: voxel dimensions: 0.3 x 0.3 x 0.6 mm; 1300 volume acquisitions; flip angle: 60°; TR/TE: 1200/18 ms; slice number: 20; matrix size: 64 x 64; field-of-view (FOV): 19.2 x 19.2 mm. GE-EPI was conducted during optogenetic OXT release. Image slice volumes were acquired in contiguous sections without interslice gap. The EPI session was followed by a high-resolution T2-weighted Rapid Imaging with Refocused Echoes (RARE) scan to image the native structural space (TR/TE: 1200/6.3 ms; matrix size: 96 x 113 x 48; voxel size: 0.2 x 0.2 x 0.3125 mm; FOV: 19.2 x 22.6 mm; RARE factor 16). A fieldmap was acquired before the EPI to correct for geometric distortion (TE1/TE2: 1.725/5.725 ms; TR: 20 ms; matrix size: 64 x 64 x 64; FOV: 20 x 20 x 20 mm).
Area of acquisition	The area of acquisition comprised the forebrain excluding brainstem and cerebellum. More posterior slices frequently had ventral signal dropout due to B0 field inhomogeneity.

Diffusion MRI ☐ Used ☒ Not used

Preprocessing

Preprocessing software	Acquired data were converted from Bruker file format to NIfTI file format, resized by a factor of 10 for better visualization, and reoriented using pvconv.pl (http://pvconv.sourceforge.net/) and a custom in-house MATLAB (Version 2020, MathWorks) routine. The first five volumes of the functional time series were removed to exclude T1 effects, leaving the remaining image volumes for further preprocessing. These data were unwarped using the presubtracted phase and magnitude field map images and realigned with a 7th degree B-spline interpolation using SPM12 (Statistical Parametric Mapping; Wellcome Department of Imaging Neuroscience; https://www.fil.ion.ucl.ac.uk/spm/) to obtain 6 rigid-body transformation parameters. fMRI time-series data were slice-time corrected (to the mean slice), and then linearly aligned to the subject's native anatomical scan. Quality checks were performed manually by visually inspecting preprocessing outputs. The 3D-anatomical data were segmented into tissue classes and a group-template in the space of the stereotactic anatomical atlas was created using Diffeomorphic Anatomical Registration Through Exponentiated Lie Algebra (DARTEL). The resulting nonlinear flow-fields were then applied to the functional images to transform them to atlas space. Spatially normalized results were visually examined for each case. The processed EPI data outputs were smoothed using a Gaussian kernel (with 0.6 mm full-width at half-maximum, fwhm).
Normalization	The 3D-anatomical data were segmented into tissue classes and a group-template in the space of the stereotactic anatomical atlas was created using Diffeomorphic Anatomical Registration Through Exponentiated Lie Algebra (DARTEL). The resulting nonlinear flow-fields were then applied to the functional images to transform them to atlas space. Spatially normalized results were visually examined for each case.
Normalization template	Allen Brain Atlas (Lein et al., Nature, 2007; Sack et al., Magn. Reson. Mater. Phys. Biol. Med., 2021)
Noise and artifact removal	The 6 realignment parameters obtained by rigid-body head motion correction were included in the GLM.
Volume censoring	We did not apply any form of volume censoring to our data.

Statistical modeling & inference

Model type and settings	For functional MRI data, in SPM12, a general linear model (fixed effects) was used, where stimulus onset times were modelled as events (stick functions) convolved with a mouse-specific HRF.
Effect(s) tested	Individual voxel-wise T-contrast maps were tested at the multi-subject level by one-sample t-test.

Specify type of analysis: ☒ Whole brain ☐ ROI-based ☐ Both

Statistic type for inference (See Eklund et al. 2016)	cluster-wise with a cluster-defining threshold of pCDT < 0.01
Correction	Family Wise Error (FWE) cluster-corrected (pFWEc < 0.05)

Models & analysis

n/a	Involved in the study
☒	☐ Functional and/or effective connectivity
☒	☐ Graph analysis
☒	☐ Multivariate modeling or predictive analysis
